# Across-cohort QC analyses of GWAS summary statistics from complex traits

**DOI:** 10.1038/ejhg.2016.106

**Published:** 2016-08-24

**Authors:** Guo-Bo Chen, Sang Hong Lee, Matthew R Robinson, Maciej Trzaskowski, Zhi-Xiang Zhu, Thomas W Winkler, Felix R Day, Damien C Croteau-Chonka, Andrew R Wood, Adam E Locke, Zoltán Kutalik, Ruth J F Loos, Timothy M Frayling, Joel N Hirschhorn, Jian Yang, Naomi R Wray, The Genetic Investigation of Anthropometric Traits (GIANT) Consortium, Peter M Visscher

**Affiliations:** 1 Queensland Brain Institute, The University of Queensland, Brisbane, Queensland, Australia; 2 School of Environmental and Rural Science, The University of New England, Armidale, New South Walsh, Australia; 3 SPLUS Game, Guangzhou, Guangdong, China; 4 Department of Genetic Epidemiology, Institute of Epidemiology and Preventive Medicine, University of Regensburg, Regensburg, Germany; 5 Medical Research Council (MRC) Epidemiology Unit, Institute of Metabolic Science, Addenbrooke’s Hospital, Cambridge, UK; 6 Department of Genetics, University of North Carolina, Chapel Hill, North Carolina, USA; 7 Channing Division of Network Medicine, Department of Medicine, Brigham and Women’s Hospital and Harvard Medical School, Boston, Massachusetts, USA; 8 Genetics of Complex Traits, University of Exeter Medical School, University of Exeter, Exeter, UK; 9 Department of Biostatistics and Center for Statistical Genetics, University of Michigan, Ann Arbor, Michigan, USA; 10 Department of Medical Genetics, University of Lausanne, Lausanne, Switzerland; 11 Institute of Social and Preventive Medicine (IUMSP), Centre Hospitalier Universitaire Vaudois (CHUV), Lausanne, Switzerland; 12 Swiss Institute of Bioinformatics, Lausanne, Switzerland; 13 The Charles Bronfman Institute for Personalized Medicine, Icahn School of Medicine at Mount Sinai, New York, New York, USA; 14 The Mindich Child Health and Development Institute, Icahn School of Medicine at Mount Sinai, New York, New York, USA; 15 The Genetics of Obesity and Related Metabolic Traits Program, Icahn School of Medicine at Mount Sinai, New York, New York, USA; 16 Department of Genetics, Harvard Medical School, Boston, Massachusetts, USA; 17 Program in Medical and Population Genetics, Broad Institute of MIT and Harvard, Cambridge, Massachusetts, USA; 18 Center for Basic and Translational Obesity Research, Boston Children's Hospital, Boston, Massachusetts, USA; 19 Division of Endocrinology, Boston Children's Hospital, Boston, Massachusetts, USA; 20 The University of Queensland Diamantina Institute, Translation Research Institute, Brisbane, Queensland, Australia; 21 A full list of members is available in the Supplementary Note.

## Abstract

Genome-wide association studies (GWASs) have been successful in discovering SNP trait associations for many quantitative traits and common diseases. Typically, the effect sizes of SNP alleles are very small and this requires large genome-wide association meta-analyses (GWAMAs) to maximize statistical power. A trend towards ever-larger GWAMA is likely to continue, yet dealing with summary statistics from hundreds of cohorts increases logistical and quality control problems, including unknown sample overlap, and these can lead to both false positive and false negative findings. In this study, we propose four metrics and visualization tools for GWAMA, using summary statistics from cohort-level GWASs. We propose methods to examine the concordance between demographic information, and summary statistics and methods to investigate sample overlap. (I) We use the population genetics *F*_st_ statistic to verify the genetic origin of each cohort and their geographic location, and demonstrate using GWAMA data from the GIANT Consortium that geographic locations of cohorts can be recovered and outlier cohorts can be detected. (II) We conduct principal component analysis based on reported allele frequencies, and are able to recover the ancestral information for each cohort. (III) We propose a new statistic that uses the reported allelic effect sizes and their standard errors to identify significant sample overlap or heterogeneity between pairs of cohorts. (IV) To quantify unknown sample overlap across all pairs of cohorts, we propose a method that uses randomly generated genetic predictors that does not require the sharing of individual-level genotype data and does not breach individual privacy.

## Introduction

To elucidate genetic architecture, which requires maximized statistical power for discovery of risk alleles of small effect, large genome-wide association meta-analyses (GWAMAs) are tending towards ever-larger scale that may contain data from hundreds of cohorts. At the individual cohort level, genome-wide association study (GWAS) analysis is often based on various genotyping chips and conducted with different protocols, such as different software tools and reference populations for imputation, inclusion of study-specific covariates and association analyses using different methods and software. Although solid quality control (QC) analysis pipelines of GWAMA exist,^1^ these analyses focus on QC for each cohort independently. With ever-increasing sizes of GWAMA, there is a need for additional QC that goes beyond the cohort-by-cohort genotype-level analysis performed to date.

In this study, we propose a new set of QC metrics for GWAMA. All these applications assume that there is a central analysis hub, where summary statistic data from GWAS are uploaded for each cohort. All methods proposed are implemented in freely available software GEAR.

## Materials and methods

### Overview of materials and methods

#### Cohort-level summary statistics

The height GWAS summary statistics were provided by the GIANT Consortium and were from 82 cohorts (174 separate files) representing a total of 253 288 individuals, and ~2.5 million autosomal SNPs imputed to the HapMap2 reference.^2^ Metabochip summary statistics for body mass index (BMI) were from 43 cohorts (120 files), representing a total of 103 047 samples from multiple ethnicities with about 200 000 SNPs genotyped on customised chips.^3, 4^

#### 1000 Genomes project samples

1000 Genomes Project (1KG) reference samples^5^ were used as the reference samples for estimating *F*_st_ and meta-PC. When assessing the global-level *F*_st_ measures, Yoruba represent African samples (YRI, 108 individuals), Han Chinese in Beijing represent East Asian samples (CHB, 103 individuals), and Utah Residents with Northern and Western European Ancestry represent European samples (CEU, 99 individuals) were employed as the reference panels. For calculating within-Europe *F*_st_, CEU, Finnish (FIN, 99 individuals), and Tuscani (TSI, 107 individuals) were employed to represent northwest, northeast, and southern Europeans, respectively. For analyses using a whole European panel, CEU, FIN, TSI, GBR (British, 91 individuals), and IBS (Iberian, 107 individuals) were pooled together as an ‘averaged’ European reference.

#### WTCCC GWAS data

WTCCC GWAS data has 2934 shared controls for seven diseases with a total of 14 000 cases.^6^ Individual GWAS was conducted for each disease using PLINK^7^ and their summary statistics used to estimate *λ*_meta_.

The four proposed metrics include:

*F*_pc_: a genome-wide comparison of allele frequency differences across cohorts or against a common reference population.
Meta-PC: principal component analysis of reported allele frequencies.
*λ*_meta_: a pairwise cohort statistic that uses allele frequency or effect size concordance to detect the proportion of sample overlap or heterogeneity.
Pseudo profile score regression: an easy to implement analysis to pinpoint each between-cohort overlapping sample that does not require the sharing of individual-level genotype data.

The technical details of these four methods summarized here can be found in the Supplementary Notes. Overview and application of these four metrics in GWAMA can be found in the Text Box.

## Results

### Population genetic QC analysis using *F*_st_

In GWAMA, only summary statistics such as allele frequencies are available to the central analysis hub, it is difficult to identify population outliers. Gross differentiation in allele frequencies at specific SNPs between GWAMA cohorts and a reference (such as 1000 Genomes Project, denoted as 1KG)^5^ are part of standard QC protocols,^1^ but checking for more differentiation than expected across the entire genome is not usually part of the QC pipeline. We propose that a genetic distance inferred from *F*_st_, which reflects genetic distance between pairwise populations, is a useful additional QC statistic to detect cohorts that are population outliers. Using the relationship between *F*_st_ and principal components,^8^ our *F*_st_ cartographer algorithm can be used to estimate the relative genetic distance between cohorts (Supplementary Notes for Method I; Supplementary Figure S1).

We applied the *F*_st_ metric to the GIANT Consortium BMI Metabochip cohorts (55 male-only cohorts, 55 female-only cohort, and 10 mixed-sex cohorts), which were recruited from multiple ethnicities,^3^ such as Europeans, African Americans in the Atherosclerosis Risk in Communities Study (ARIC) and cohorts from Jamaica (SPT), Pakistan (PROMISE), Philippines (CLHNS), and Seychelles (SEY). For each Metabochip cohort, we sampled 30 000 independent markers to calculate *F*_st_ values with each of three 1KG samples (CEU, CHB, and YRI, respectively). For validation of the method, we also calculated *F*_st_ values against the 1KG Japanese (JPT, Japanese in Tokyo, Japan), Indian (GIH, Gujarati Indian in Houston, US), Kenyan (LWK, Luhya in Webuye, Kenya), and European samples (IBS, Iberian populations, Spain; FIN, Finnish, Finland; TSI, Toscani, Italy, and GBR, British in England, and Scortland, GBR), to see whether the known genetic origins of those cohorts can be recovered.

**Figure 1:**
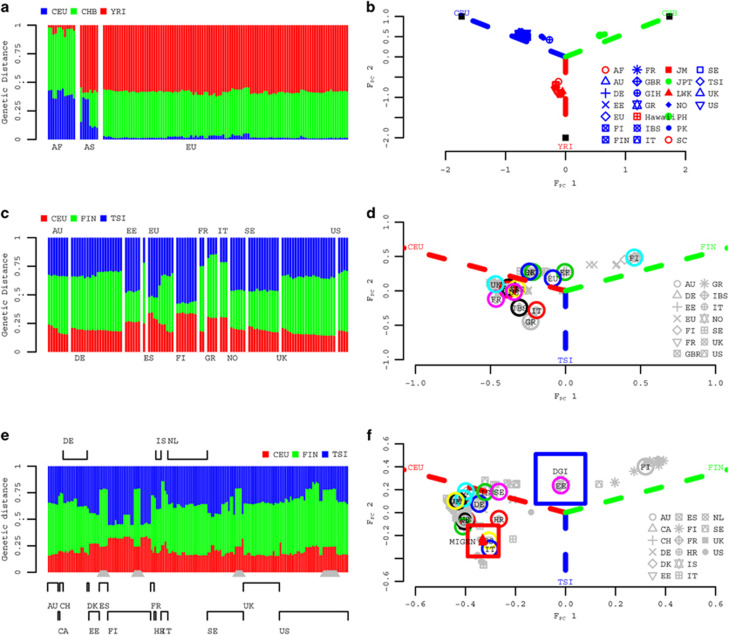
Recovery of cohort-level genetic background and inference of their geographic locations for GIANT BMI Metabochip cohorts and GIANT GWAS height cohorts using the *F*_st_-derived genetic distance measure. (**a**) Genetic distance spectrum for all Metabochip cohorts to CEU, CHB, and YRI. The origins of the cohorts are denoted on the horizontal axis. (**b**) Projection for the Metabochip cohorts into *F*_PC_ space defined by YRI, CHB, and CEU reference populations. The *x* and *y* axis represent relative distances derived from the genetic distance spectrum. Three dashed lines, blue for CEU, green for CHB, and red for YRI, partitioned the whole *F*_PC_ space to three genealogical subspaces. (**c**) The genetic distance spectrum for the Metabochip European cohorts to CEU – northwest Europeans, FIN – northeast European, and TSI – southern Europeans. The nationality of the cohorts is denoted on the horizontal axis. (**d**) The projection for the Metabochip European cohorts to the *F*_PC_ space defined by CEU, FIN, and TSI reference populations. The whole space is further partitioned into three subspaces, CEU-TSI genealogical subspace (red and blue dashed lines), FIN-TSI genealogical subspace (green-blue dashed lines), and CEU-FIN genealogical subspace (red-green dashed lines), respectively. (**e**) Each cohort has three *F*_st_ values by comparing with CEU, FIN, and TSI reference samples. The height of each bar represents its relative genetic distance to these three reference populations. The nationalities of the cohorts are denoted along the horizontal axis. The grey triangles along the *x* axis indicate MIGEN cohorts. (**f**) Given the three *F*_st_ values, the location of each cohort can be mapped. The whole space was partitioned into three subspaces, CEU-TSI genealogical subspace (red and blue dashed lines), FIN-TSI genealogical subspace (green and blue dashed lines), and CEU-FIN genealogical subspace (red and green dashed lines). DGI (in the blue box) had samples from the Botnia study. Across the MIGEN cohorts (denoted as red triangles in the red box), the same allele frequencies (likely calculated from a South European cohort) were presented for each cohort. The open circles represent the mean of inferred geographic locations for the cohorts from the same country. Cohort/country codes: AF, African; AU, Australia; CA, Canada; CH, Switzerland; DE, Germany; DK, Denmark; EE, Estonia; ES, Iberian Population in Spain in 1KG; EU, European Nations; FI, Finland; FIN, Fins in 1000 Genomes Project (1KG); FR, France; GBR, British in 1KG; GIB, Gujarati Indian in 1KG; GR, Greece; Hawaii, Hawaii in USA; IBS, Iberian Population in Spain in 1KG; IT, Italy; IS, Iceland; JM, Jamaica; JPT, Japanese in 1KG; LWK, Luhya in 1KG; NL, Netherlands; NO, Norway; PH, the Philippines; PK, Pakistan; SC, Seychelles; SCT, Scotland; SE, Sweden; TSI, Tuscany in 1KG; UK, United Kingdom; US, United States of America.

According to the origins of the samples, each Metabochip cohort showed a different genetic distance spectrum to the three reference populations (Figure 1a). The JPT and Philippine cohorts had very small genetic distances to CHB, as expected, but large to CEU and YRI; however, the Pakistan cohorts showed much closer genetic distances to CEU than to CHB and YRI, indicating their demographic history. The cohorts sampled from Jamaica, Seychelles, Hawaii, and the African American ARIC cohort had small genetic distances to YRI, but large distances to CHB and CEU. For most European cohorts, as expected, the distances to CEU were very small compared with those to CHB and YRI. Given their relative distances to CEU, CHB, and YRI, using our *F*_st_ cartographer algorithm (Supplementary Notes for Method I; Supplementary Figure S1), the cohorts were projected into a two-dimensional space, called *F*_st_-derived principal components (*F*_PC_) space, constructed by YRI, CHB, and CEU as the reference populations (Figure 1b). The allocation of the cohorts to the *F*_PC_ space resembles that of eigenvector 1 against eigenvector 2 in principal component analysis (PCA), and is similar to those observed in PCA using individual-level GWAS data for populations of various ethnicities such as in 1KG samples.^5^ Therefore, our method to place cohorts in geographical regions from GWAS summary statistics works well at a global-population scale.

We next investigated whether our genetic distance method works at a much finer geographic scale. It is known that using individual-level data, PCA can mirror the geographic locations for European samples.^9^ Here we analyzed the 103 GIANT European-ancestry Metabochip cohorts (48 male-only cohorts, 47 female-only cohorts, and 8 mix-sex cohorts) for fine-scale *F*_st_ genetic distance measure using the CEU, FIN, and TSI reference populations, which represent northwest, northeast, and southern European populations, respectively. For each of the GIANT European-ancestry Metabochip cohorts, *F*_st_ was calculated relative to each of these three reference populations and showed concordance with the known origin of the samples (Figure 1c). For example, cohorts from Finland and Estonia were close to FIN but distant to TSI; cohorts from South Europe such as Italy and Greece had small genetic distance to TSI; and cohorts from West Europe had small genetic distance to CEU. Similarly, the projected origin for each European-ancestry Metabochip cohort resembles its geographic location within the European map as expected (Figure 1d). Therefore, *F*_pc_ based upon population differentiation also works at a fine scale.

We next applied the *F*_st_ genetic distance measures to 174 GIANT height GWAS cohorts (79 male-only cohorts, 76 female-only cohorts, and 19 mixed-sex cohorts; excluding Metabochip data), which were all of European ancestry imputed to the HapMap reference panel.^2^ Given the three *F*_st_ values to CEU, FIN, and TSI (Figure 1e), the geographic origin for each cohort can be inferred as for the GIANT BMI Metabochip data. The projected coordinates of each GWAS cohort matches its origin very well (Figure 1f). For example, a Canadian cohort, the Quebec Family Study (QFS), was closely located to DESIR, a French cohort, consistent with the French genetic heritage of the QFS.^10^ In addition, we also observe complexity due to mixed samples from different countries. For example, the DGI/Botnia study had samples recruited from Sweden and Finland, and its inferred geographic location is in between of the Swedish cohorts and Finnish cohorts.^11^ We also note that for the Myocardial Infarction Genetics Consortium (MIGEN) cohorts, which are recruited from Finland, Sweden, Spain, and the United States, the same allele frequencies were reported for all their sub-cohorts, and all cohorts were allocated to southern Europe (very closely located to 1KG IBS cohort; Figure 1f and Supplementary Figure S2). As the allele frequencies, used in QC steps to eliminate low-quality loci, were not directly used in estimating genetic effects in the GWAMA, the reported allele frequencies in MIGEN have not impacted much on the published GWAMA results.^2^

Next, we show that *F*_st_ can detect populations that have a different demographic past. Using all 1KG European samples as the reference panel (eg, an ‘averaged’ European reference panel), most cohorts in GIANT had *F*_st_<0.005 with this average, which agrees with previously reported results using individual-level data from European nations.^9^ A few cohorts showed large *F*_st_, such as the AMISH cohort with *F*_st_=0.018, and the North Swedish Population Health Study^12^ with *F*_st_=0.014. Both populations are known to have been genetically isolated (Supplementary Figure S3).

### PCA for allele frequencies (meta-PCA)

Given the same allele frequencies as used for *F*_st_-based analysis above, we conducted PCA for allele frequencies, denoted as meta-PCA (or mPC). In meta-PCA, each cohort was analogously considered as an ‘individual’. For example, 120 Metabochip cohorts were considered as a sample of 120 ‘individuals’. Although the inferred ancestral information was for each cohort rather than any individuals, implementation of meta-PCA was the same as the conventional PCA (Supplementary Notes for Method II). Meta-PCA was tested with 1KG samples. It indicated that meta-PCA could reveal the genetic background for each cohort as precisely as that based on individual-level data (Supplementary Figure S4).

We applied meta-PCA to 120 Metabochip cohorts for nearly 34K common SNPs between Metabochip and 1KG variants, with the inclusion of 10 1KG cohorts (East Asian: CHB and JPT; South Asian: GIH; European: CEU, FIN, GBR, IBS, and TSI; African: LWK and YRI) as the reference cohorts. Consistent with demographic information, the inferred ancestral information of each cohort agreed well with demographic information. For example, PROMISE (Pakistan) located very close to GIH, CLHNS (Philippines) close to CHB and JPT, ARIC (African American) and SPT (Jamaican) close to YRI and LWK, and the European cohorts close to CEU and FIN (Figure 4a).

We also applied meta-PCA to 174 GIANT height GWAS cohorts for nearly 1M SNPs, with the inclusion of 10 1KG reference cohorts. At the global-population level, the 174 cohorts were all allocated close to CEU and FIN, consistent with their reported demographic information (Figure 4b). For fine-scale inference, we conducted meta-PCA again but with the inclusion of the five 1KG European samples. As demonstrated (Figure 4c), the resolution of the inferred relative location between European cohorts reflected their real geographical locations, as previously observed using individual-level data.^9^ For example, of the four cohorts from Italy, the MICROS cohort was from South Tyrol, northern Italy. MICROS had its meta-PC coordinates much closer to CEU than another three Italian cohorts, reflecting its geographic location; the InCHIANTI cohort had its coordinates almost identical to TSI; the cohort SardiNIA located more southward than TSI, reflecting its relative geographic and genetic isolation as recently confirmed.^13^ Similarly, in the sub-plots for Finland and Sweden, the cohorts from the MIGEN consortium, which all had reported allele frequencies of south Europe origin, were located near 1KG TSI and IBS.

These results were consistent to what was observed from *F*_pc_ as described in the last section, and also agreed well with demographic information. Therefore, based on the reported allele frequencies, the demographic information could be verified by the meta-PCA method.

### *λ*_meta_ to detect pairwise cohort heterogeneity and sample overlap

In this study, we use the summary statistics for a pair of cohorts to calculate *λ*_meta_, a metric that examines heterogeneity from the concordance of reported effect sizes and sampling variance. For a SNP marker (*i*), given its reported estimated effect size (*b*_*i*_) and sampling variance (*σ*_*i*_^2^) in a pair of cohorts 1 and 2, we can calculate a test statistic 
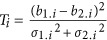
, the ratio between the squared difference of their reported effects and the sum of their reported sampling variances. We constructed 30 000 *T* statistics using markers in linkage equilibrium along the genome for a pair of cohorts. Under the null hypothesis of no overlapping samples/heterogeneity, *T* follows a *χ*^2^ distribution with 1 degree of freedom (Supplementary Notes for Method III).

Analogous to *λ*_GC_, 
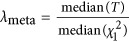
, the ratio between the median of the 30 000 *T* values and the median of a *χ*^2^ statistic with 1 degree of freedom (a value of 0.455) has an expected value of 1 for two independent GWAS summary statistics sets for the same trait. When there is heterogeneity between estimated genetic effects, the expectation is *λ*_meta_>1, and in contrast *λ*_meta_<1 if there are overlapping samples. In general, not only overlapping samples but also close relatives present in different cohorts can lead to correlated summary statistics generating *λ*_meta_<1. However, unless the proportion of overlapping relatives is substantial and their phenotypic correlation is high, the correlation of the summary statistics due to the effective number of overlapping samples (*n*_o_) is expected to be dominated by the same individuals contributing phenotypic and genetic information to different cohorts (Supplementary Figure S5). Furthermore, if genomic control is applied to adjust the sampling variance, then *λ*_meta_ will be reduced relative to its value without genomic control for *λ*_GC_.

GWAS summary statistics for schizophrenia were available in two phases: the first had 9394 controls and 12 462 cases,^14^ and in the next phase ~18 000 Swedish samples were added.^15^ Such a substantial overlap sample between these two sets of summary statistics led to the estimated value of *λ*_meta_ as low as 0.257 (Supplementary Figure S6), consistent with this known overlap. In contrast, heterogeneity between data sets (represented by *λ*_meta_>1) was observed between GWAS summary statistics of rheumatoid arthritis from European and Asian studies,^16^ for which *λ*_meta_=1.09 (Supplementary Figure S7). In addition, we note that the distribution of the empirical *T*-statistics deviates from expectation at the upper tail of the distribution, suggesting differences in effect size or linkage disequilibrium between these two ancestries.

**Figure 2:**
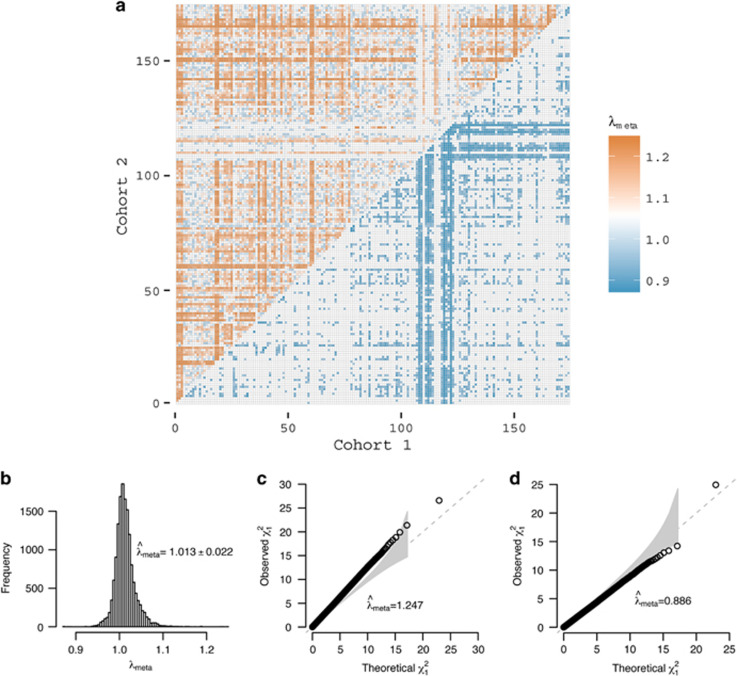
*λ*_meta_ for the GIANT height GWAS cohorts. (**a**) Given 174 cohorts, there are 15 051 *λ*_meta_ values, which provide the overview of the quality control of the summary statistics. The heat map represents 15 051 *λ*_meta_ statistics, and the *x* and *y* axis index each pair of cohorts. The pairs of cohorts showed heterogeneity (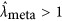) are illustrated on left-top triangle, and homogeneity (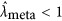) on right-bottom triangle. (**b**) The distribution of *λ*_meta_ from 174 cohorts/files used in the GIANT height meta-analysis. The overall mean of 15 051 *λ*_meta_ is 1.013, and SD is 0.022. (**c**) Illustration for homogeneity between two cohorts (SORBS MEN and WOMEN), *λ*_meta_=0.876. (**d**) Illustration of SardiNIA and WGHS, this pair of cohorts has *λ*_meta_=1.245. The grey band represents 95% confidence interval for *λ*_meta_.

Next, we estimated *λ*_meta_ from pairs of cohorts from the 174 GIANT height GWAS cohorts.^2^ We found no evidence for substantial sample overlap but do observe between-cohort heterogeneity and technical artifacts. From the 174 GIANT height GWAS,^2^ we calculated 15 051 cohort pairwise *λ*_meta_ values, resulting in a bell-shaped distribution (Figure 2a and b), with the mean of 1.013 and the empirical SD of 0.022, which was greater than theoretical SD of 0.014. The empirical mean and SD can be used to construct a *z*-score test for each *λ*_meta_. These results are consistent with a small amount of heterogeneity, which is not unexpected due to variation of actual (unknown) genetic architecture and analysis protocols. However, the mean is close to 1.0 and based upon this QC metric, the results are consistent with stringent QC and data cleaning. The minimum *λ*_meta_ value was ~0.88 (between SORBS men and SORBS women; Figure 2c), with *P*-value<1e−10 (testing for the difference from 1), and the maximum was 1.245 (between SardiNIA and WGHS; Figure 2d), with *P*-value<1e−10, leading to the most deflated and inflated *λ*_meta_ across GIANT height study cohorts, both were significant after correction for multiple testing. Of note, SORBS were analyzed using a method that corrected for relatedness, which potentially led to the deflated *λ*_meta_ as implicated by the theory (Supplementary Notes for Method III). Illustrating *λ*_meta_ (Figure 2a) highlighted that 20 cohorts from the MIGEN consortium showed substantially lower *λ*_meta_ with many other cohorts (right-bottom triangle in Figure 2a) than the average, consistent with over-conservative models for statistical association analyses being used in these cohorts – which may be due to very small sample size (ranging from 36 to 320 for the 20 MIGEN cohorts, with an average sample size of 132). Consistent with this, cohorts from MIGEN also have many of their *λ*_GC_<1 (Supplementary Figures S8 and S9). In contrast, the SardiNIA cohort (4303 samples) showed heterogeneity with nearly all other cohorts (Supplementary Figures S8 and S9), perhaps due to unknown artifacts or a slightly different genetic architecture for height as result of demographic history.^17^

The statistical power of detection of overlapping samples is maximized when a pair of cohorts has equal sample size (Supplementary Figure S10), or in other words the confidence interval for null hypothesis of no overlapping samples depends on the sample sizes for a pair of cohorts. As a comparison, the estimation of a correlation between the genetic effects for a pair of cohorts has been proposed to quantify overlapping samples,^18, 19^ but this metric is confounded with genetic architecture, such as heritability underlying the trait(s) (Table 1; Supplementary Notes IV). When there was heritability, the estimated correlation between genetic effects could be biased and could lead to an incorrect inference about overlapping samples for a pair of cohorts. When there was no heritability, the estimated correlation was correct and agreed well with the one estimated with *λ*_meta_. As existence of heritability is one of the reasons to perform GWAMA, so *λ*_meta_ is preferred when estimating overlapping samples between cohorts.

Another parameterization of *λ*_meta_ is to estimate it from differences in allele frequencies between a pair of cohorts instead of differences between estimated effect sizes (Supplementary Notes III; Supplementary Figure S11).

### Detection of overlapping samples using pseudo profile score regression

In many circumstances, individual cohorts are not permitted to share individual-level data, either by national law or by local ethical review board conditions. Although the metric *λ*_meta_ can be transformed to give an estimate of *n*_o_ between cohorts for quantitative traits, it cannot give an estimate of overlapping samples in case–control studies due to the ratio of the cases and controls in each study. To get around this problem, Turchin and Hirshhorn^20^ created a software tool, Gencrypt, which utilizes a security protocol known as one-way cryptographic hashes to allow overlapping participants to be identified without sharing individual-level data. We propose an alternative approach, pseudo profile score regression (PPSR), which involves sharing of weighted linear combinations of SNP genotypes with the central meta-analysis hub. In essence, multiple random profile scores are generated for each individual in each cohort, using SNP weights supplied by the analysis hub, and the resulting scores are provided back to the analysis hub. PPSR works through three steps (Supplementary Notes for Method IV; Supplementary Figure S12), and the purpose of PPSR is to estimate a relationship-like matrix of *n*_*i*_ × *n*_*j*_ dimension for a pair of cohorts, which have *n*_*i*_ and *n*_*j*_ individuals, respectively. Each entry of the matrix is filled with genetic similarity for a pair of samples from each of the two cohorts, estimated via the PPSR. The central hub analysts can determine the best set of SNPs that each individual analysis hub uses to generate PPS. Without the loss of generality, a set of loci directly genotyped in all cohorts would make good candidate set of SNPs for PPS.

We use WTCCC data as an illustration to detect 2934 shared controls between any two of the diseases by PPSR. Among 330K not palindromic loci, we randomly picked *M*=100, 200, and 500 SNPs, to generate pseudo profile scores. It generated 21 cohort-pair comparisons, leading to the summation for 488 587 090 total individual-pair tests. To have an experiment-wise type I error rate=0.01, type II error rate=0.05 (power=0.95) for detecting overlapping individuals, we needed to generated at least 57 PPSs. We generated scores *S*=[*s*_1_,*s*_2_,*s*_3_,…,*s*_57_], where each *s* is a vector of *M* elements, sampled from a standard normal distribution. *S* is shared across seven cohorts for generating PPSs for each individual. In total, 57 PPSs were generated for each individual in each cohort. For a pair of cohorts, PPSR was conducted for each possible pair of individuals for any two cohorts over the generated PPSs. Once the regression coefficient (*b*) was greater than the threshold, here *b*=0.95, the pair of individuals was inferred to be having highly similar genotypes, implying that the individual was included in both cohorts (Supplementary Notes for Method IV).

**Figure 3:**
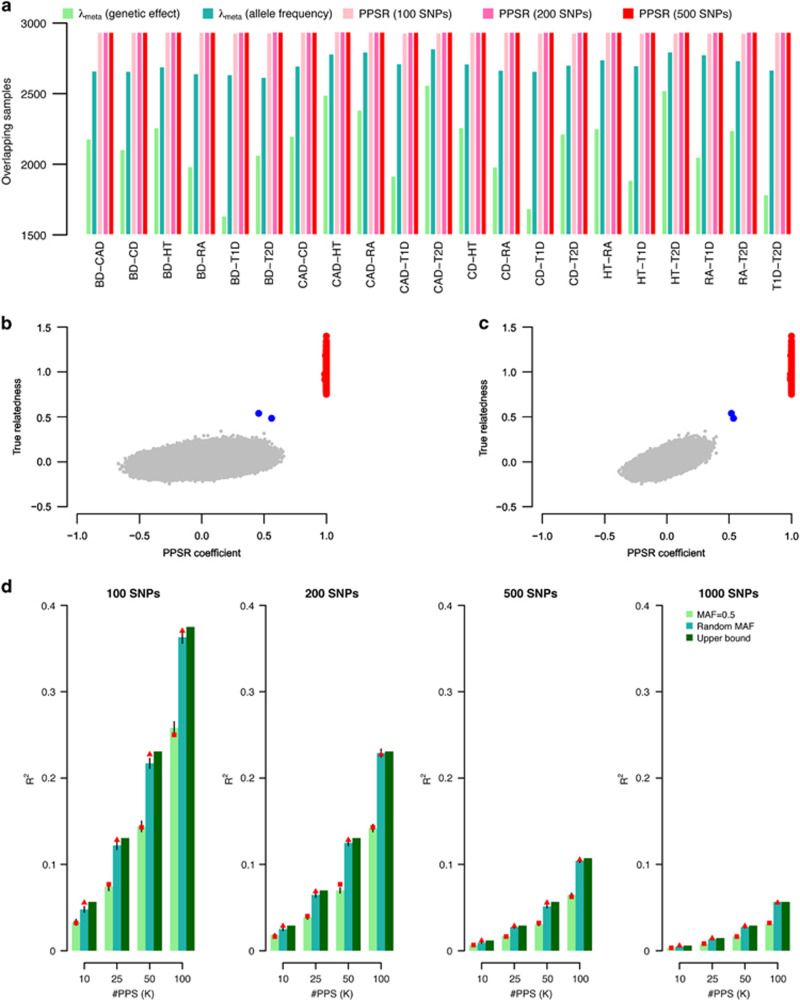
Pseudo profile score regression for pinpointing overlapping samples/relatives. (**a**) Each cluster represents a pair of cohorts as denoted on the *x* axis. Within each cluster, from left to right, the detected overlapping controls using *λ*_meta_ based either on effect size estimates or minor allele frequency (MAF), PPRS using 100, 200, and 500 markers. WTCCC cohort codes: BD for bipolar disorder, CAD for coronary artery disease, CD for Crohn’s disease, HT for hypertension, RA for rheumatoid arthritis, T1D for type 1 diabetes, and T2D for type 2 diabetes. (**b)** Illustration for regression coefficients between WTCCC BD and CAD from 57 pseudo profile scores (PPS) generated from 500 markers. The *x* axis is the PPSR regression coefficients and *y* axis is real genetic relatedness (as calculated from individual-level genotype data). The red points are the shared controls between two cohorts, and blue points are first-degree relatives. (**c**) The PPS regression coefficients for detecting overlapping first-degree relatives using 286 PPS generated from 500 markers. (**d)** Decoding genotypes from the PPS. Given the set of profile scores, one may run a GWAS-like analysis to infer the genotypes. The ratio between the number of markers (*M*) and number of pseudo profile scores (*K*) determines the potential discovery of individual-level information. The higher the ratio and, the higher the allele frequency, the less information can be recovered. From left to right, the profile scores generated using different number of markers. The *y* axis is a *R*^2^ metric representing the accuracy between the inferred genotypes and the real genotypes. From left to right panels, 100, 200, 500, and 1000 SNPs were used to generate 10, 20, 50, and 1000 profiles scores. In each cluster, the three bars are inferred accuracy using different MAF spectrum alleles, given the SE of the mean.

**Figure 4:**
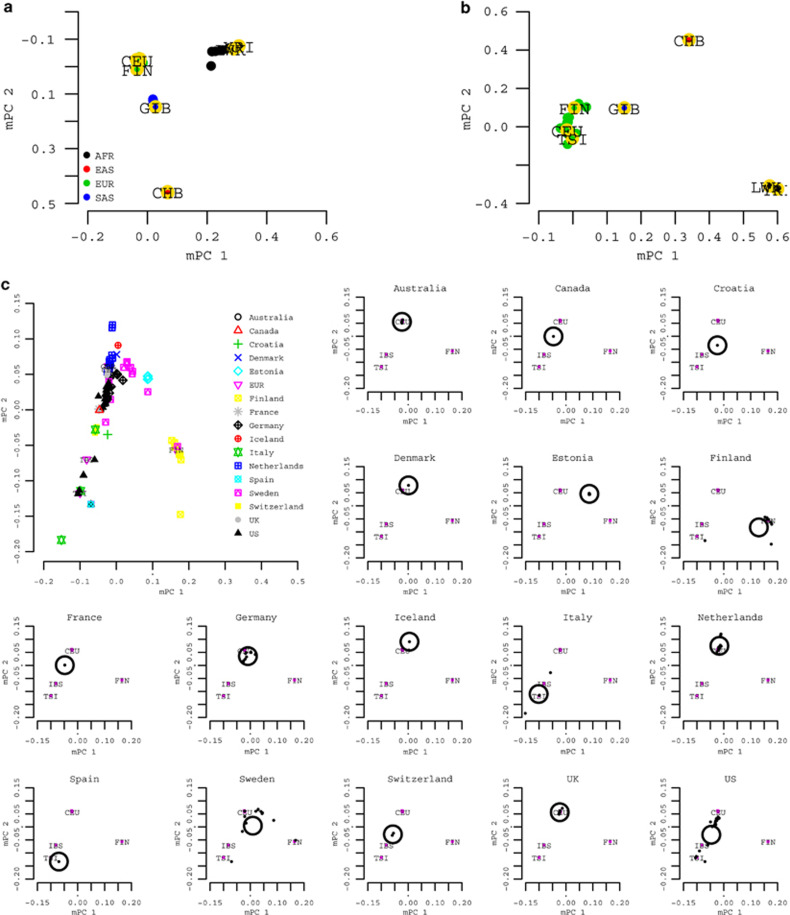
Recovery of cohort-level genetic background for GIANT cohorts using meta-PCA. (**a**) The recovery of cohort-level genetic background using meta-PCA analysis for GIANT BMI Metabochip cohorts. The *x* and *y* axis represent the first two eigenvectors from meta-PCA. In meta-PCA, Metabochip cohorts could be classified into African ancestry (AFR), European ancestry (EUR), East Asian Ancestry (EAS), and South Asian Ancestry (SAS). (**b**) The recovery of cohort-level genetic background using meta-PCA analysis for GWAS height cohorts. The *x* and *y* axis represent the first two eigenvectors inferred from meta-PCA (mPC). The genetic background inferred with the inclusion of ten 1000 Genomes reference populations. The 1000 Genome cohorts, yellow open circles, were added for comparison in **a**–**c**. The genetic background and relative geographic location for 174 GIANT height cohorts. The large plot on top left was an overview of 174 cohorts, and the rest of plots were classified by the reported demographic information of cohorts. Within each country-level plot, the small black points represent one cohort, and the large open circle the mean coordinates for those cohorts from the same country.

When using 200 and 500 random SNPs, all the known 2934 shared controls were detected from 21 cohort-pairwise comparison; when using 100 randomly SNPs, on average 2931 shared samples were identified, which is more accurate than using *λ*_meta_ constructed using either genetic effects or allele frequencies (Figure 3a). In addition, for detected overlapping samples, there were no false positives observed – consistent with simulations that show the method was conservative in the controlling type I error rate (Supplementary Notes for Method IV). For comparison, we also used the Gencrypt to detect overlapping samples using the same set of SNPs as used in PPSR. Although Gencrypt guidelines suggest use of at least 20 000 random SNPs,^20^ selecting 500 random SNPs in the WTCCC cohorts also provided good accuracy with Gencrypt, and on average about 2920 (99.6% of the shared controls) overlapping samples were detected, only slightly lower than PPSR. For example, for BP and CAD, Gencrypt detected 2912 shared controls, but was unable to identify ~20 overlapping controls, due to missing data (on average 1% missing rate).

Furthermore, PPSR is able to detect pairs of relatives. For example, between the BD and CAD cohorts, two pairs of apparent first-degree relatives were detected (Figure 3b). To find additional first-degree relatives between BD and CAD cohorts, at least 265 PPSs were required to have a type I error rate of 0.01 and type II error rate of 0.05 for a regression coefficient cutoff of 0.45, a threshold for first-degree relatives. As expected, all other individuals that did not show high relatedness did not reach the threshold of 0.45 of the PPS regression coefficient for first-degree relatives (Figure 3c). Gencrypt did not detect any first-degree relatives.

PPSR for each individual uses very little personal information and can be minimized so that there is very low probability of decoding it. One way to attempt to decode the genotypes from PPS is to reverse the PPSR, so that the individual genotypes can be predicted in the regression (Supplementary Notes for Method IV). The individual-level genotypic information that can be recovered by an analyst, who knows the *S* matrix (the weights for generating PPS), is determined by the ratio between the number of markers (*M*) that generated PPS and the number of PPS (*K*). Therefore, inferred information on individual genotypes can be minimized and tailored to any specific ethics requirements. We suggest 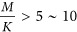 to protect the privacy with sufficient accuracy (Figure 3d).

## Discussion

In this study, we provide four metrics for monitoring and improving the quality of large-scale GWAMA based on summary statistics. Using the *F*_st_-derived genetic distance measure, we can place all cohorts on an inferred geographic map and can easily identify cohorts that are genetic outliers or that have unexpected ancestry. In application, we should note that the *F*_st_ measure can identify unusual summary information, such as detected in the MIGEN cohorts from GIANT Consortium GWAMAs, in which the same allele frequencies were reported for all cohorts. Meta-PCA can also be used to infer the genetic background of cohorts. The high concordance between *F*_pc_ and meta-PCA indicates the both methods are robust.

In practice, meta-PCA is much easier to implement when there are many cohorts, but *F*_PC_ that has close-form analytical results provides a theoretical ground for meta-PCA. There are limitations for both *F*_PC_ and meta-PCA. First, *F*_PC_ depends on the choice of reference cohorts, such as 1KG reference cohorts, and the projection may be slightly different when other reference cohorts are adopted. Resembling any PCA, the projection from meta-PCA depends on the context of all cohorts, and the inclusion or exclusion of other cohorts will change the projection slightly. However, we believe the impact will not influence the inference of the genetic background of cohorts in a meta-analysis. Second, various mechanisms can give an identical projection in PCA. The purpose of both methods is to find the discordance between demographic information and genetic information, or outliers, in GWAMA.

Our third metric *λ*_meta_ provides information on sample overlap and heterogeneity between cohorts by utilizing the estimated allelic effect sizes and their standard errors. In most meta-analyses, the overall *λ*_meta_ is likely to be slightly >1 solely due to unknown heterogeneity, slight as observed, in generating the phenotype and genotype data that cannot be accounted for by QC. The observed mean of *λ*_meta_ for the GIANT height GWAMA was 1.03 but with more variation than expected by chance. The strong correlation between *λ*_GC_ and *λ*_meta_ indicated the reported sampling of the reported data were systematically driven by analysis protocols, such as single-marker regression and linear mixed model methods. For cohorts with *λ*_GC_<1 and *λ*_meta_<1, it is likely that the GWAS modeling strategy employed for GWAS in the cohort was too conservative, eg, MIGEN cohorts might have on average too small sample size for each cohort. Conversely, for cohorts with *λ*_GC_>1 and *λ*_meta_>1, results are too heterogeneous, perhaps reflecting systematically smaller sampling variances of the reported genetic effects. As GWAMA often uses inverse-variance-weighted meta-analysis,^21^ such cohorts may lead to incorrect weights to the different cohorts in the meta-analysis, suggesting that the statistical analysis in meta-analyses can be improved by applying better weighting factors.

It is well recognised that overlapping samples may inflate the type-I error rate of GWAMA and therefore lead to false positives. Although *post hoc* correction of the test statistic is possible,^18, 19, 21^ stringent QC ruling out overlapping samples makes the whole analysis easier and lowers the risk of false positives. A better solution would be to rule out shared samples at the start, for pairs of cohorts that show deflated *λ*_meta_, and we propose PPSR to accomplish this.

## Figures and Tables

**Table 1:** The estimated correlation for a pair of cohorts via their summary statistics given 30 000 independent loci

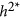	n_*1,2*_	n_*1*_	n_*2*_	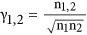	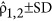	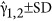
0.25	100	1000	1000	0.1	0.1072±0.0064	0.101±0.0093
		1000	2000	0.0707	0.0814±0.0054	0.0709±0.0088
		1000	5000	0.0447	0.0615±0.0055	0.0425±0.0096
		1000	10 000	0.0316	0.0556±0.0063	0.0325±0.0099
0.25	1	1000	1000	0.001	0.0092±0.0056	0.0017±0.0093
		1000	2000	0.0007	0.0126±0.0053	0.0006±0.0079
		1000	5000	0.000447	0.0189±0.0060	0.0016±0.0090
		1000	10 000	0.000316	0.0259±0.0059	0.0008±0.0092
0	100	1000	1000	0.1	0.0996±0.0052	0.094±0.0085
		1000	2000	0.0707	0.0704±0.0048	0.0712±0.0097
		1000	5000	0.0447	0.0453±0.0057	0.0441±0.0090
		1000	10 000	0.0316	0.0335±0.0057	0.0325±0.0079

Notes: Heritability was simulated on 1000 QTLs. We also tried 100 QTLs, and results were nearly identical; *n*_1_, *n*_2_, and *n*_1,2_ represent the sample size for cohort 1, 2, and overlapping samples between them. *γ*_1,2_ represents the true correlation for a pair of summary statistics due to overlapping samples. 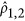 represents the estimated correlation estimated via direct correlation between summary statistics, the method proposed by Bolormaa *et al.*^18^ and Zhu *et al.*^19^. 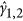 represents the estimated correlation estimate via *λ*_meta_, 
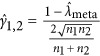
.

## Genome-wide association meta-analysis QC in a nutshell

**Metric 1:** ***F***_**st**_**-based inference of cohort origins**

*F*_st_ reflects genetic relatedness for cohorts, and consequently can be used to infer or confirm the genetic origins of cohorts. For example, the PROMISE cohort, which is from Pakistan, had its global-level coordinates between CEU (Europeans) and CHB (east Asians), and was very close to GIH (Indians), which agrees with the demographic history of the Pakistani population (Figure 1).

**Metric 2: meta-PCA inference of cohort origins**

Meta-PCA resembles conventional genotype-based PCA^8^, but the input data are the reported allele frequencies of cohorts rather than individual genotypes, hence summary statistics. Once a cohort has a projection that disagrees with its demographic history, the central meta-analysis hub should contact the individual cohort manager for clarification (Figure 4).

For metrics 1 and 2, the inferred cohort origins can be projected at a global or regional level. For example, CEU, YRI, and CHB can be chosen as global reference populations, and CEU, FIN, and TSI chosen as within-Europe reference populations.

**Diagnosis:** The central meta-analysis hub is suggested to implement either metric 1 or metric 2 once the summary statistics are received. These two metrics can rule out major errors such as incorrectly generated summary statistics (such as wrong reference allele frequencies) or incorrectly uploaded files.

**Metric 3:** ***λ***_**meta**_ **for detecting unusual sampling properties for a pair of cohorts.**

*λ*_meta_ examines a pair of cohorts for: (1) *E*(*λ*_meta_)=1 if a pair of cohorts is consistent by being samples drawn from the same population; (2) *E*(*λ*_meta_)<1 if a pair of cohorts is too similar, such as due to overlapping samples; (3) *E*(*λ*_meta_)>1, if a pair of cohorts is too dissimilar, such as due to different data analysis protocols or difference in genetic architecture. Summary statistics are often generated from different protocols, which introduce technical heterogeneity and push the empirical  is often slightly >1 (Figure 2b). For statistical test for overlapping samples between any pair of cohorts, its *z*-score can be constructed as , in which  can be estimated from all possible pairs of *λ*_meta_ between the cohorts in a GWAMA. As demonstrated, an outlier cohort often generates a special pattern in the distribution of *λ*_meta_ (see the blue bands at the off-diagonal space in Figure 2a).

The central meta-analysis hub can use the distribution/heat map of *λ*_meta_ to monitor the abnormality of the reported genetic effects and potential overlapping samples. In addition, *λ*_meta_ for all pairs of cohorts makes a correlation matrix, which can be integrated into generalized meta-analysis for the correction of overlapping samples.^21^

**Diagnosis**: If a cohort is observed to have  with many other cohorts, it may reflect other systematic issues, such as accidentally *λ*_GC_ correction is implemented, but should not, for the raw summary statistics. If a cohort has  with many other cohorts, it may reflect a very different protocol is used in generating the summary statistics, or may reflect its very different genetic architecture.

**Metric 4: PPSR for pinpointing overlapping samples**

When *λ*_meta_ provides strong evidence for a proportion of overlapping samples between a pair of cohorts, PPSR can further pinpoint the exact overlapping individuals/relatives included in these two cohorts. PPSR is better to solve an unreasonable small value *λ*_meta_ between a pair of cohorts, say *λ*_meta_<0.8, and its implementation should be coordinated by the central analyst, who can balance the quality of the summary statistics and identification concerns (Figure 3d).

## Supplementary Material

Supplementary Figures

Supplementary Notes

Supplementary Tube-Video
