# Supplementary material for: Across-cohort QC analyses of GWAS summary statistics from complex traits
Source: Eur J Hum Genet. 2016 Aug 24;25(1):137–46. doi: 10.1038/ejhg.2016.106 (PMC5159754; doi:10.1038/ejhg.2016.106)

New QC metrics for GWAS meta analysis: Supplementary Figures

Across-cohort QC analyses of GWAS summary statistics from complex traits

Authors: Guo-Bo Chen[1], Sang Hong Lee[1,2], Matthew R Robinson[1], Maciej Trzaskowski[1], Zhi-Xiang Zhu[3], Thomas W Winkler[4], Felix R Day[5], Damien C Croteau-Chonka[6,7], Andrew R Wood[8], Adam E Locke[9], Zoltán Kutalik[10-12], Ruth J F Loos[13-15], Timothy M Frayling[8], Joel N Hirschhorn[16-19], Jian Yang[1,21], Naomi R Wray[1], The Genetic Investigation of Anthropometric Traits (GIANT) Consortium[20], Peter M Visscher[1,21]

Affiliations:
[1] Queensland Brain Institute, The University of Queensland, Brisbane, Queensland, Australia
[2] School of Environmental and Rural Science, The University of New England, Armidale, New South Walsh, Australia
[3] SPLUS Game, Guangzhou, Guangdong, China
[4] Department of Genetic Epidemiology, Institute of Epidemiology and Preventive Medicine, University of Regensburg, Regensburg, Germany
[5] Medical Research Council (MRC) Epidemiology Unit, Institute of Metabolic Science, Addenbrooke's Hospital, Cambridge, UK
[6] Department of Genetics, University of North Carolina, Chapel Hill, North Carolina, USA
[7] Channing Division of Network Medicine, Department of Medicine, Brigham and Women's Hospital and Harvard Medical School, Boston, Massachusetts, USA
[8] Genetics of Complex Traits, University of Exeter Medical School, University of Exeter, Exeter, UK
[9] Department of Biostatistics and Center for Statistical Genetics, University of Michigan, Ann Arbor, Michigan, USA
[10] Department of Medical Genetics, University of Lausanne, Lausanne, Switzerland
[11] Institute of Social and Preventive Medicine (IUMSP), Centre Hospitalier Universitaire Vaudois (CHUV), Lausanne, Switzerland
[12] Swiss Institute of Bioinformatics, Lausanne, Switzerland
[13] The Charles Bronfman Institute for Personalized Medicine, Icahn School of Medicine at Mount Sinai, New York, New York, USA
[14] The Mindich Child Health and Development Institute, Icahn School of Medicine at Mount Sinai, New York, New York, USA
[15] The Genetics of Obesity and Related Metabolic Traits Program, Icahn School of Medicine at Mount Sinai, New York, New York, USA
[16] Department of Genetics, Harvard Medical School, Boston, Massachusetts, USA
[17] Program in Medical and Population Genetics, Broad Institute of MIT and Harvard, Cambridge, Massachusetts, USA
[18] Center for Basic and Translational Obesity Research, Boston Children's Hospital, Boston, Massachusetts, USA
[19] Division of Endocrinology, Boston Children's Hospital, Boston, Massachusetts, USA
[20] A full list of members is available in the **Supplementary Note**
[21] The University of Queensland Diamantina Institute, Translation Research Institute, Brisbane, Queensland, Australia

Correspondence should be addressed to

GBC (chenguobo@gmail.com) or PMV (peter.visscher@uq.edu.au)

New QC metrics for GWAS meta analysis: Supplementary Figures

Supplementary Figures

Figure S1 Demonstration for Fst **cartographer algorithm.** 3

Figure S2 Inference for geographic locations for GIANT height GWAS cohorts with the inclusion of MIGEN. 4

Figure S3 Fst **for GIANT height cohorts using 1000 Genome European as reference.** 5

Figure S4 Comparison between Meta-PCA and genotype PCA on 1000 Genome samples using nearly 1M SNPs. 6

Figure S5 λmeta **and the effective number of overlapping samples** (no). 7

Figure S6 λmeta **for PGC schizophrenia summary statistics.** 8

Figure S7 λmeta **for rheumatoid arthritis summary statistics between Eueropean samples and Asian samples.** 9

Figure S9 λmeta **and** λgc **for GIANT height GWAS cohorts.** 11

Figure S10 Statistical power for detecting overlapping samples between a pair of cohorts given type I error rate of 0.05. 13

Figure S11 Using λmeta **constructed either on genetic effects or on allele frequency to estimate overlapping samples between WTCCC 7 diseases.** 14

Figure S12 Workflow for PPSR regression. 15

New QC metrics for GWAS meta analysis: Supplementary Figures

Figure S1 Demonstration for F_{st} cartographer algorithm.

a) For example, for FTC cohort from Finland, its F_{st} to CEU=0.010, F_{st} to FIN = 0.0051, F_{st} to TSI=0.0157. So a point cutting the CEU-FIN edge into 0.010:0.0051 is found first, and similarly to find the points on another two edges. Connecting the three points made FTC triangle. The gravity of the FTC triangle is the inferred geographic coordinates for FTC.
The algorithm can be applied to b) SardiNIA from Italy, and c) B58C cohort from England, to find their coordinates. d) A coarse map can be constructed.

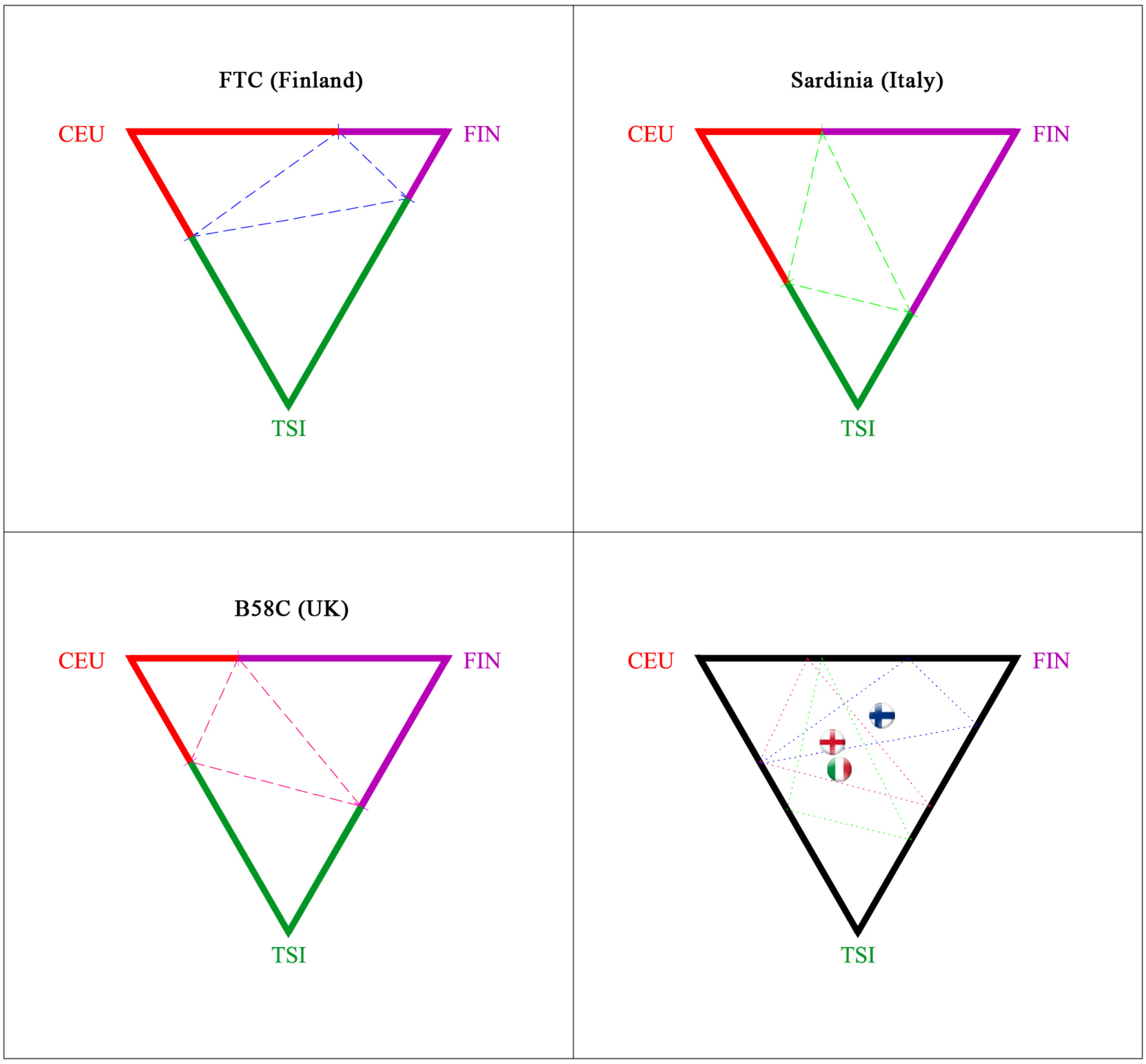

		F_{st} to the reference samples		
Cohort	Nation	CEU	FIN	TSI
FTC.WOMEN	Finland	0.010	0.0051	0.0157
SARDINIA.ALL	Italy	0.0017	0.00286	0.00138
B58C-WTCCC	England	0.0021	0.0052	0.0040

Figure S2 Inference for geographic locations for GIANT height GWAS cohorts with the inclusion of MIGEN.

The open circles represent the mean of the inferred geographic locations for the cohorts from the same country. Red triangles (near ES circle – the 1000 Genomes Project IBS cohort) represent MIGEN cohorts, which are from Sweden, Finland, USA, and Spain. However, they are all filled with the same allele frequencies in the summary statistics uploaded to the data sever.

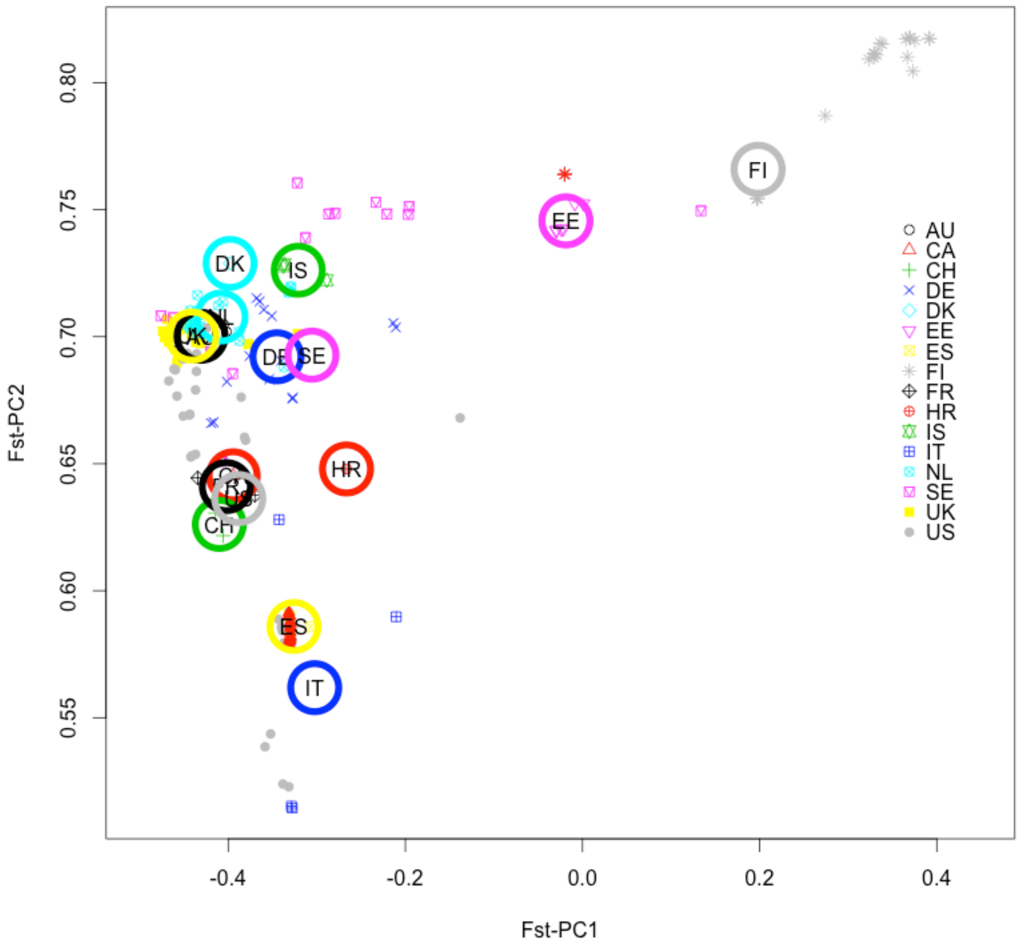

Figure S3 F_{st} for GIANT height cohorts using 1000 Genome European as reference.

Using all 1000 Genomes Project European samples as the reference panel – an “averaged” European population, F_{st} is calculated for each cohort. Amish population (from US) and NSPHS population (from Sweden) shows high F_{st}. Cohorts from the same nations were arranged together on the x-axis.

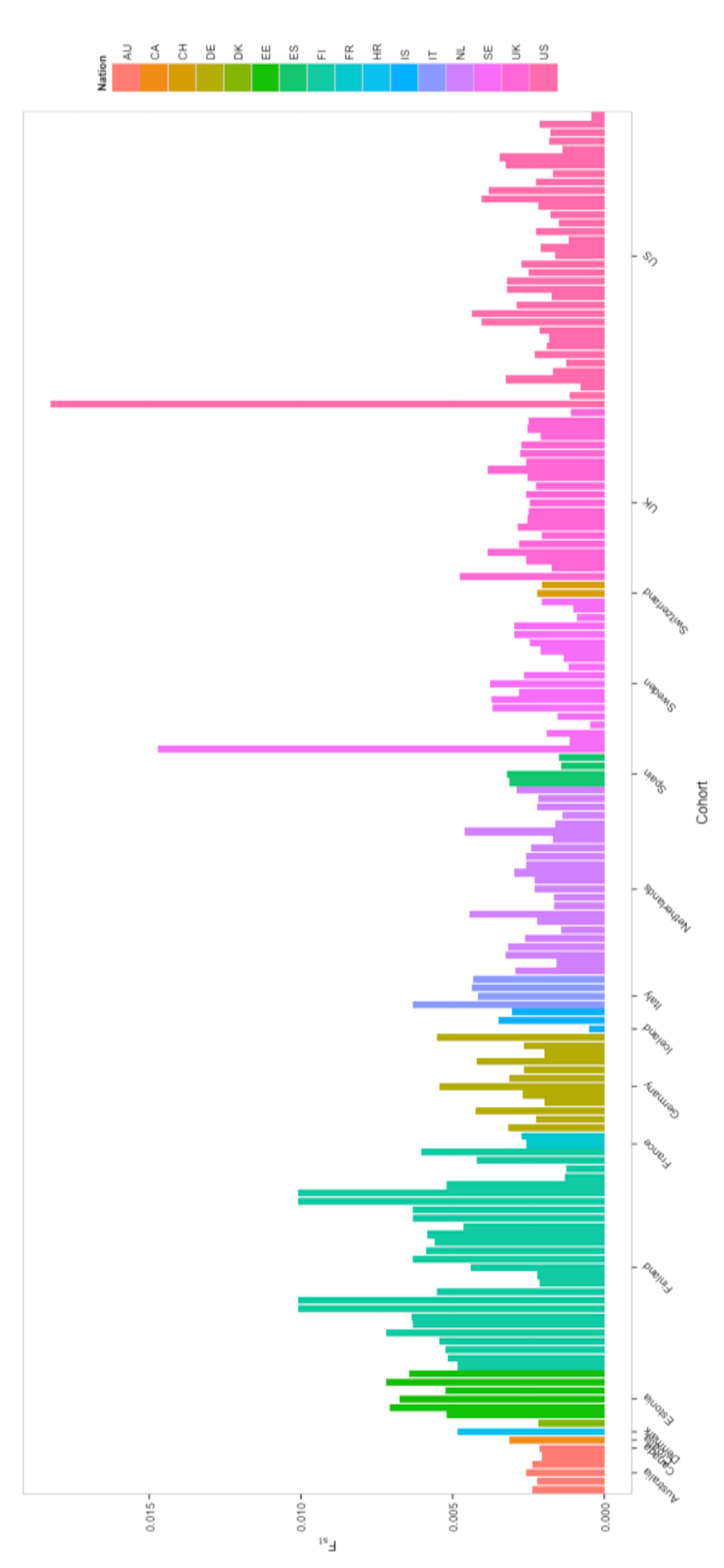

Figure S4 Comparison between Meta-PCA and genotype PCA on 1000 Genome samples using nearly 1M SNPs.

The cohort-level allele frequencies were estimated for 26 1KG cohorts, and meta-PCA was conducted. **(a)** The projection of cohorts based on cohort-level allele frequency for 1KG samples on the first two eigenvectors. **(b)** Conventional PCA based on individual genotypes on the first two eigenvectors, the mean of the 1KG individuals within each cohort is represented with the big circles. **(c)** The correlation, measured in R^2, between meta-PCA and genotype PCA for the first twenty eigenvectors.
The projected cohorts were consistent with their genetic origins. In contrast, conventional PCA was also conducted on 1KG individual genotypes directly, and the mean coordinates for each cohort was then calculated. As illustrated, these two techniques resulted in nearly identical projection for 1KG, and the correlation between cohort coordinates remained consistently high, $R^2 > 0.9$, for the first eight eigenvectors.

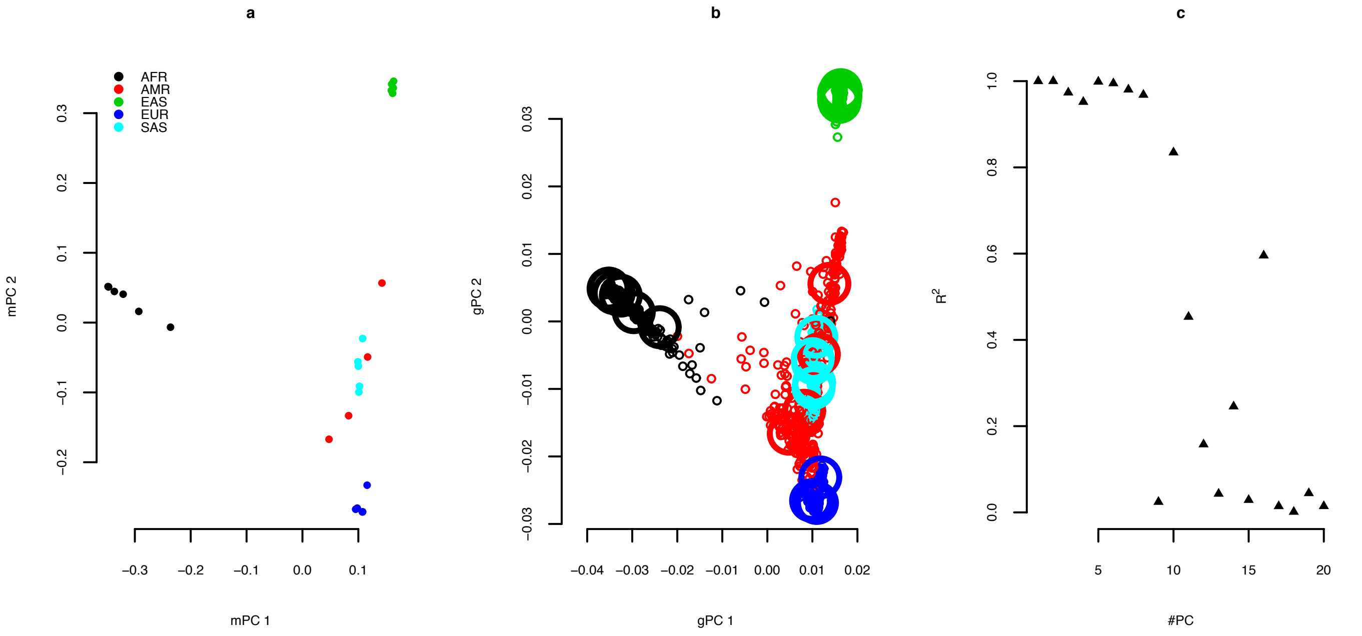

New QC metrics for GWAS meta analysis: Supplementary Figures

Figure S5 $\boldsymbol{\lambda_{meta}}$ and the effective number of overlapping samples ($\boldsymbol{n_o}$).

Two cohorts, with 1000 individuals each and 30,000 independent loci are simulated. The genetic effects are estimated using single-marker association. Using these 30,000 summary statistics, the $\boldsymbol{\lambda_{meta}}$ is computed for each locus and contrasted to the null distribution. Each vertical panel represents different heritability, and each horizontal panel represents different combination of overlapping samples. The red line, with its expected slope of $(1 - \frac{n_o}{n_1+n_2})$, in each plot represents the expected distribution of the sampled loci, and gray area represents the 95% confidence interval for $\boldsymbol{\lambda_{meta}}$ under the null distribution. The overlapping first-degree relatives cause correlation between summary statistics when heritability is large (first two horizontal panels). The overlapping samples always cause reduced $\boldsymbol{\lambda_{meta}}$ (the last two horizontal panels).

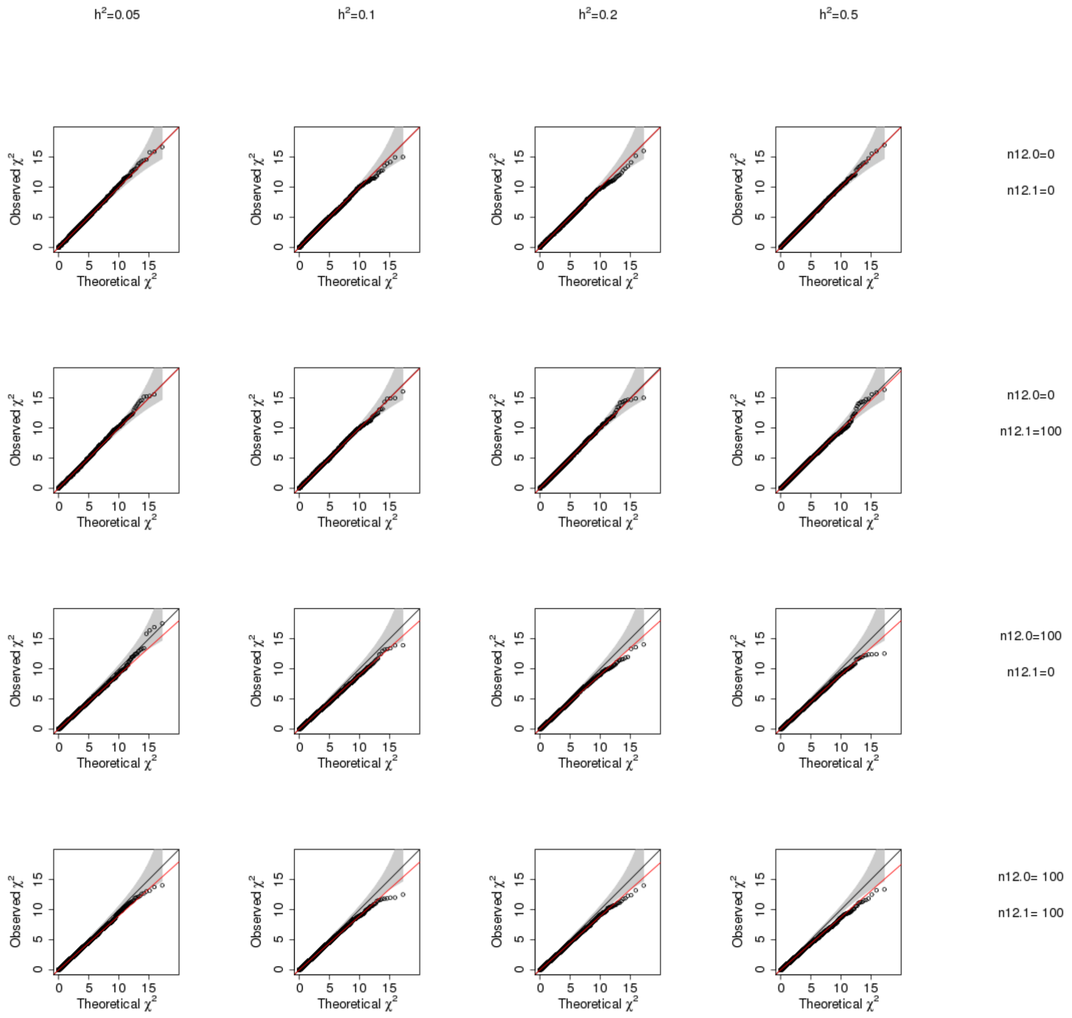

Figure S6 λ_{meta} for PGC schizophrenia summary statistics.

There is substantial sample overlap between PGC1 and PGZ+Swedish, and led to $\lambda_{meta} = 0.257$.

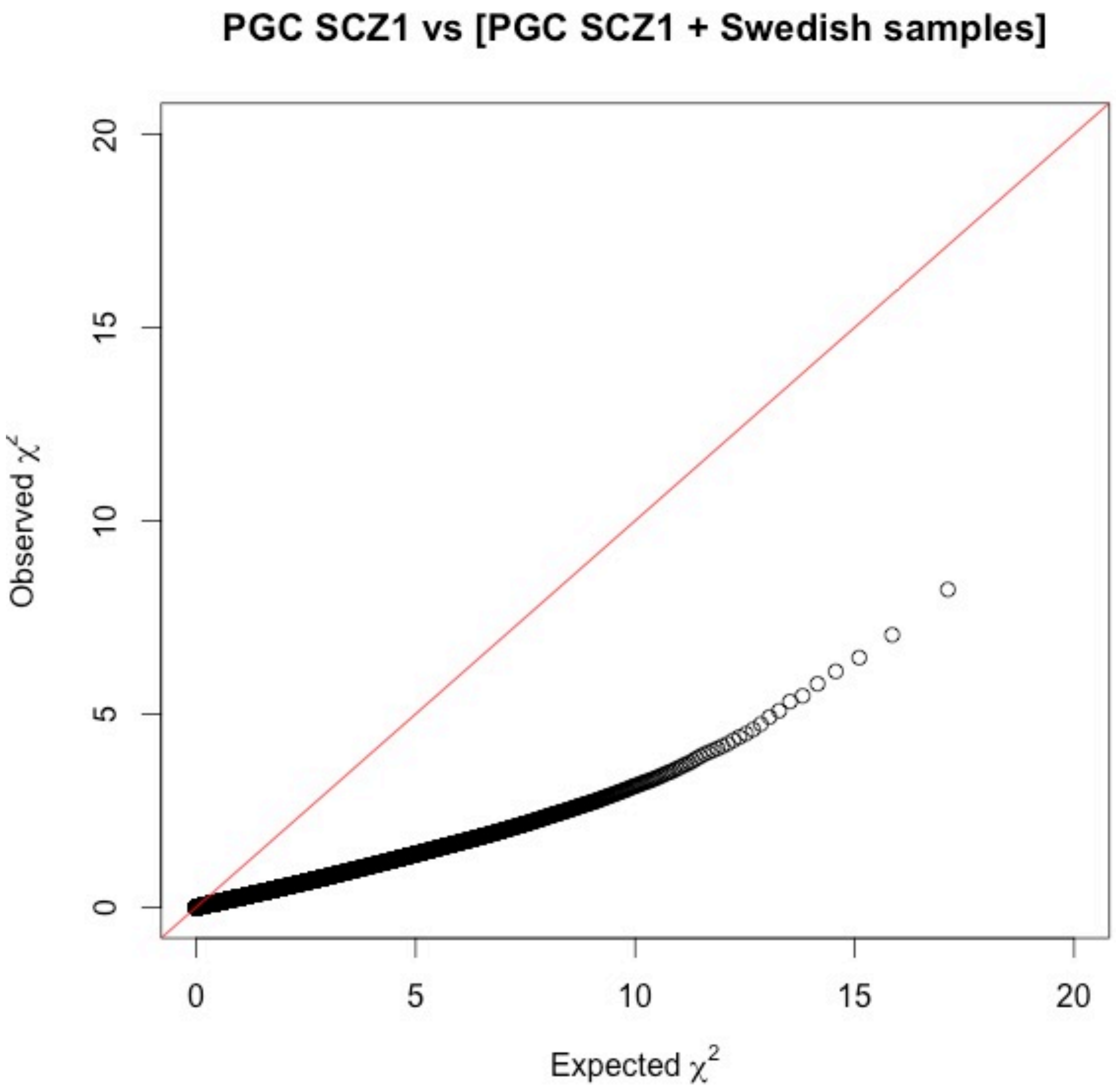

Figure S7 λ_{meta} for rheumatoid arthritis summary statistics between Eueropean samples and Asian samples.

Genetic architecture may lead to inflated $\lambda_{meta} = 1.07$ from RA GWAS summary statistics.

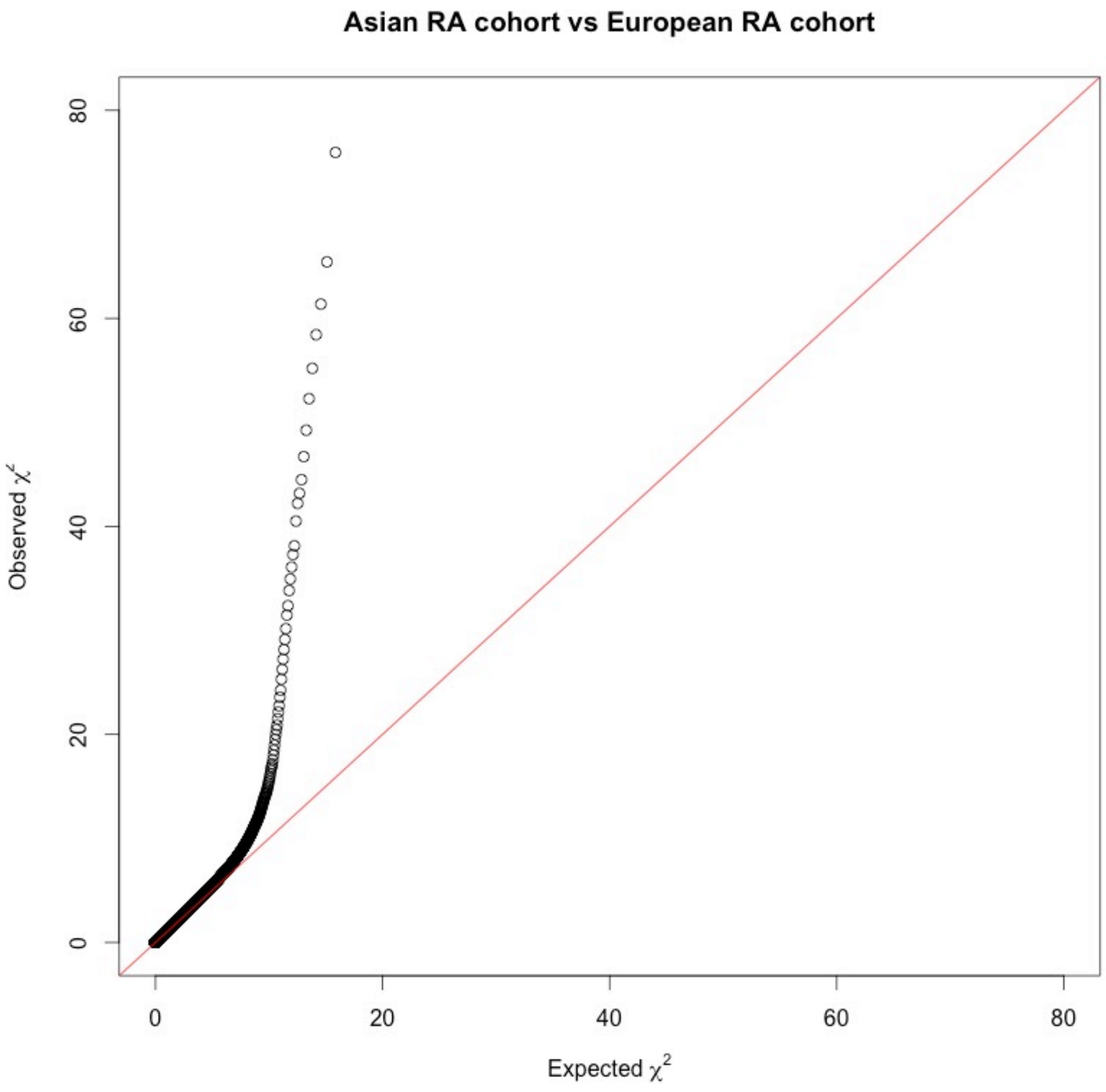

Figure S8 No correlation between $\boldsymbol{\lambda_{meta}}$ and $\boldsymbol{\bar{\lambda}_{GC}}$ for summary statistics when there are no technical errors. For a single cohort, $\lambda_{GC} = \frac{b_1^2}{\omega\sigma_1^2}/0.456$, in which ω is an unknown factor (technical artifact) of value around 1. When $\omega = 1$, there is no technical error, the behaviour of λ_{GC} as previous described (Yang et al., 2011): proportional to sample size under polygenic model.

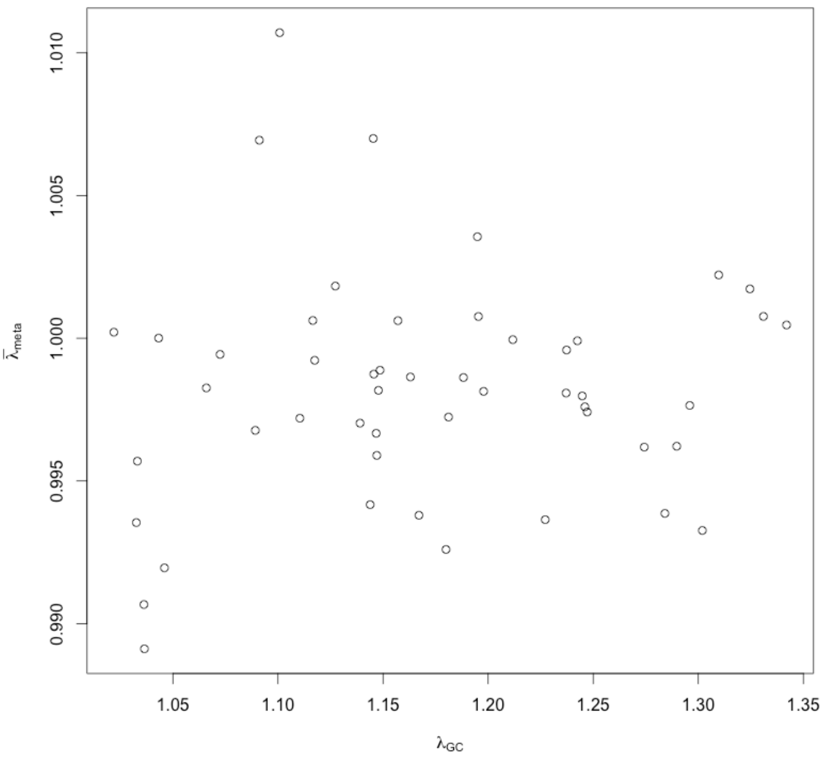

Figure S9 $\bar{\lambda}_{meta}$ and λ_{gc} for GIANT height GWAS cohorts.

(a) Sample size of each cohort against λ_{GC}. The linear regression is presented as a dashed line, λ_{GC} = 1.021 + 0.0000033N (N is reported sample size), and $R^2 = 0.013$. **(b)** Sample size of each cohort against $\bar{\lambda}_{meta}$, which was the mean of a cohort's λ_{meta} over all other cohorts. The linear regression is presented as a dashed line, $\bar{\lambda}_{meta}$ = 1.012 + 0.00000055N, and $R^2 = 0.055$. **(c)** λ_{GC} against $\bar{\lambda}_{meta}$ for each cohort, showing a strong correlation, $R^2 = 0.70$. The black dash line indicates the regression slope for all 174 pairs: $\bar{\lambda}_{meta}$=0.7251+0.281λ_{GC}+e. The red dashed line indicates the regression slope for 20 pairs of MIGEN cohorts: $\bar{\lambda}_{meta}$=0.369+0.631λ_{GC}+e. The side of each circle is proportional to sampling size on logarithm scale. **(d)** Small sample size leads to a correlation between $\bar{\lambda}_{meta}$ and λ_{GC} using 174 GIANT height GWAS sample size. 30,000 independent loci, minor allele frequency ranged from 0.1~0.5, were simulated, and $h^2 = 0.5$. The red dashed line indicates the regression slope for 20 simulated MIGEN cohorts, $\bar{\lambda}_{meta} = 0.488 + 0.510\lambda_{GC}$+e ($R^2 = 0.78$). The side of each circle is proportional to sampling size on logarithm scale. **(e)** λ_{meta} for whole MIGEN to 174 cohorts. 20 MIGEN files were combined together to make "whole MIGEN" via meta-analysis, and the summary statistics were used to calculate λ_{meta} with 174 cohorts using 30,000 independent loci. As MIGEN cohorts were part of "whole MIGEN", their λ_{meta} were in general below 1. The dashed line is the mean of λ_{meta} of the "whole MIGEN". The subplot (red box) shows a strong correlation of 0.93 between λ_{meta} (for "whole MIGEN" vs each MIGEN cohort), and sample size of each MIGEN cohort.

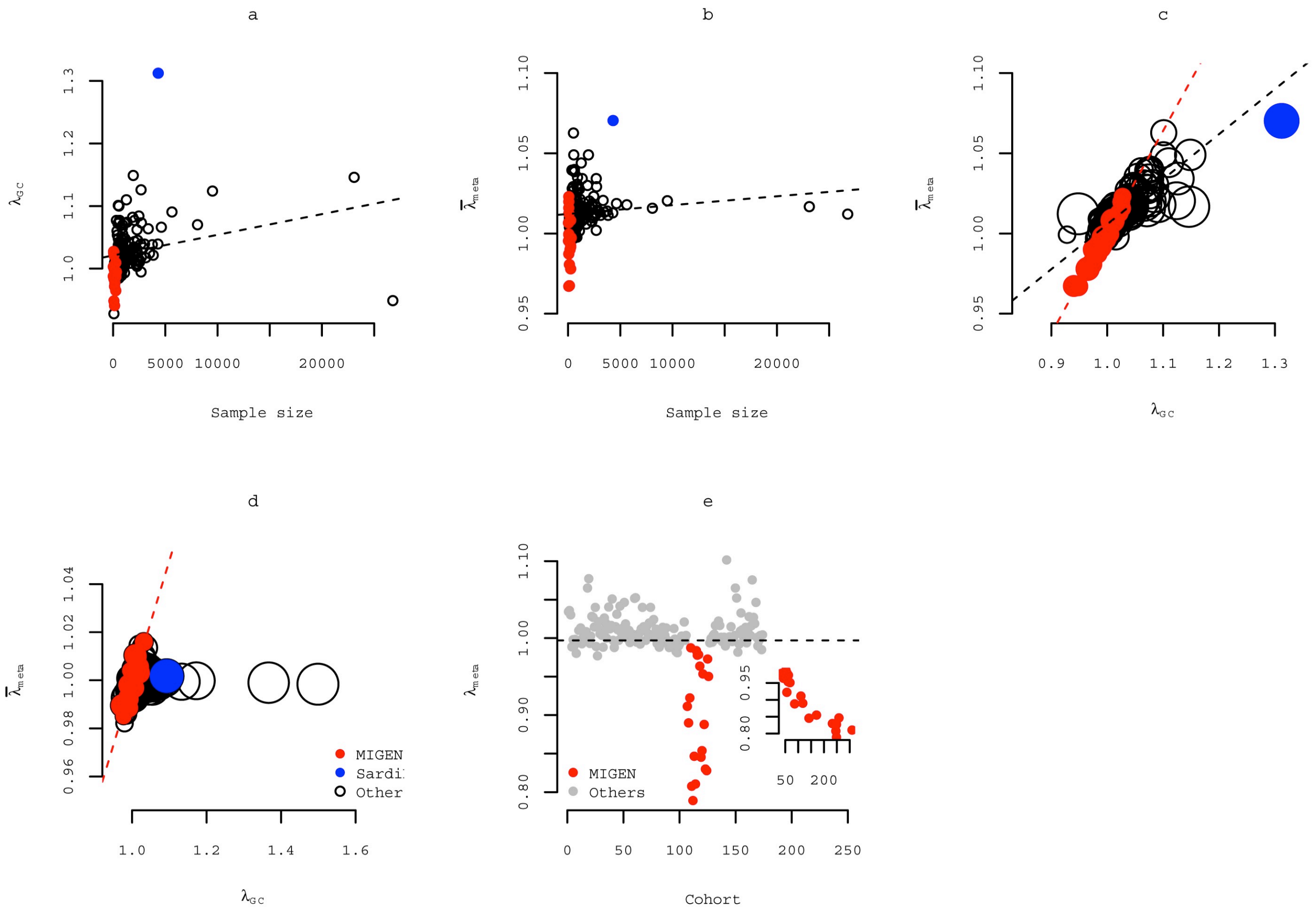

We investigated the relationship between $\bar{\lambda}_{meta}$ (the mean of all λ_{meta} values of a given cohort with each of the other 173 GIANT height cohorts) and λ_{GC} among the GIANT height cohorts. If there are no technical issues, such as inflated or deflated sampling variance for the estimated effects, we would expect to see: i) a correlation between λ_{GC} and sample size; ii) no correlation between $\bar{\lambda}_{meta}$ and sample size; iii) no correlation between $\bar{\lambda}_{meta}$ and λ_{GC}. Consistent with a previous study[25], for a polygenic trait such as height λ_{GC} of each cohort was related to its sample size (correlation of 0.235, p = 0.0018). In contrast, the

correlation between $\bar{\lambda}_{meta}$ and sample size was of 0.116 (p = 0.127) (**Figure S9a,b**). Nevertheless, the correlation between the mean of $\bar{\lambda}_{meta}$ and λ_{GC} was 0.836 (p<10e-16) for 174 GIANT height cohorts (**Figure S9c**). We note that the 20 MIGEN cohorts had proportionally small λ_{GC} and $\bar{\lambda}_{meta}$, with very high correlation between them ($\rho = 0.98$); in contrast, the SardiNIA cohort, which had the largest λ_{GC}, showed the largest $\bar{\lambda}_{meta}$ (1.070 ± 0.049), standing out as a special case among the GIANT height cohorts. Assuming a polygenic model of $h^2 = 0.5$ over 30,000 independent loci, we simulated 174 cohorts using the actual size samples from the GIANT height cohorts, and observed an increased correlation ($R^2 = 0.78$) between $\bar{\lambda}_{meta}$ and λ_{GC} for simulated cohorts with sample sizes of the MIGEN cohorts **(Figure S9d)**. Other effects, such as inflated/deflated sampling variance of the estimated genetic effects could also lead to correlation between $\bar{\lambda}_{meta}$ and λ_{GC} (**Supplementary Figure S8**). In addition, we constructed a single MIGEN analysis by combining the 20 MIGEN cohorts using an inverse variance weighted meta-analysis[26], and calculated λ_{meta} between this combined MIGEN cohort and all 174 cohorts. As expected, the combined MIGEN had $\lambda_{meta} = 0.90 \pm 0.07$ with 20 MIGEN cohorts due to overlapping samples. In contrast, $\lambda_{meta} = 1.01 \pm 0.02$ with 154 other cohorts, was consistent with neither heterogeneity nor sample overlap. Given that the MIGEN (2,340 samples) and SardiNIA (4,303 samples) cohorts contributed less than 3% of the total sample size (253,288 samples from the GIANT height GWAS cohorts), any impact of unusual λ_{meta} values on the meta-analysis results is very small.

Simulated cohort-level summary statistics for this figure. M independent loci were generated for cohort-level summary statistics. Each locus had allele frequency p_i, which was sampled from a uniform distribution ranging from 0.1 to 0.5, and had genetic effect b_i, sampled from a standard normal distribution $N(0,1)$. After rescaling, $\Sigma_{i=1}^{M} 2p_i(1-p_i)b_i^2 = h^2$. p and b were treated as true parameters. For a particular cohort with n samples, its $\tilde{p}_i \sim N(p_i, \frac{p_i(1-p_i)}{2n})$, $\tilde{b}_i \sim N(b_i, \frac{1}{2np_i(1-p_i)})$, and the sampling variance for $\tilde{b}_i$ is $\sigma_{b_i}^2 = \frac{1}{2n_i p_i(1-p_i)}$. All cohorts were assumed to share common genetic architecture, and differences were only due to genetic drift, allele frequencies and sampling variance of genetic effects.

Figure S10 Statistical power for detecting overlapping samples between a pair of cohorts given type I error rate of 0.05.

The y-axis represents statistical power, and the x-axis the number of overlapping samples. Cohort 1 has 1,000, 5,000, 10,000, or 25,000 samples, and cohort 2 has 1,000 samples. The two cohorts have 25, 50, 100, 200, and 500 overlapping samples. Bottom panel: the corresponding 95% confidence interval is given for each scenario in the top panel. The statistical power is maximized when the two cohorts have the same sample size.

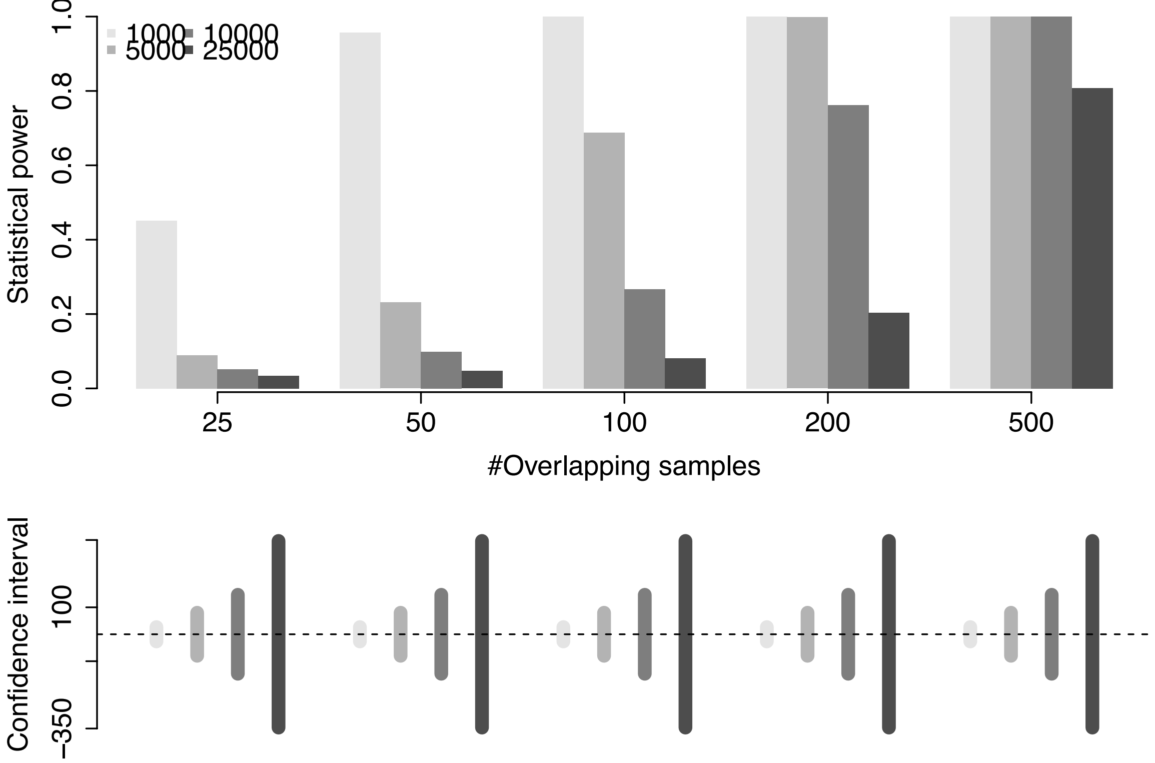

Figure S11 Using λ_{meta} constructed either on genetic effects or on allele frequency to estimate overlapping samples between WTCCC 7 diseases.

λ_{meta} can be constructed on reported genetic effects (red bars), and alternatively can be constructed on allele frequency (blue bars). Both can be used to detecting overlapping samples. The x-axis indicate the pair of cohorts in WTCCC, and the y-axis represent the estimated overlapping samples based on λ_{meta}, which is estimated over 30,000 markers. 95% confidence interval is represented on top of each bar. The mean of the estimated overlapping samples using λ_{meta} on genetic effects is 2127.38±257.73, for λ_{meta} on MAF is 2707.99±58.41.

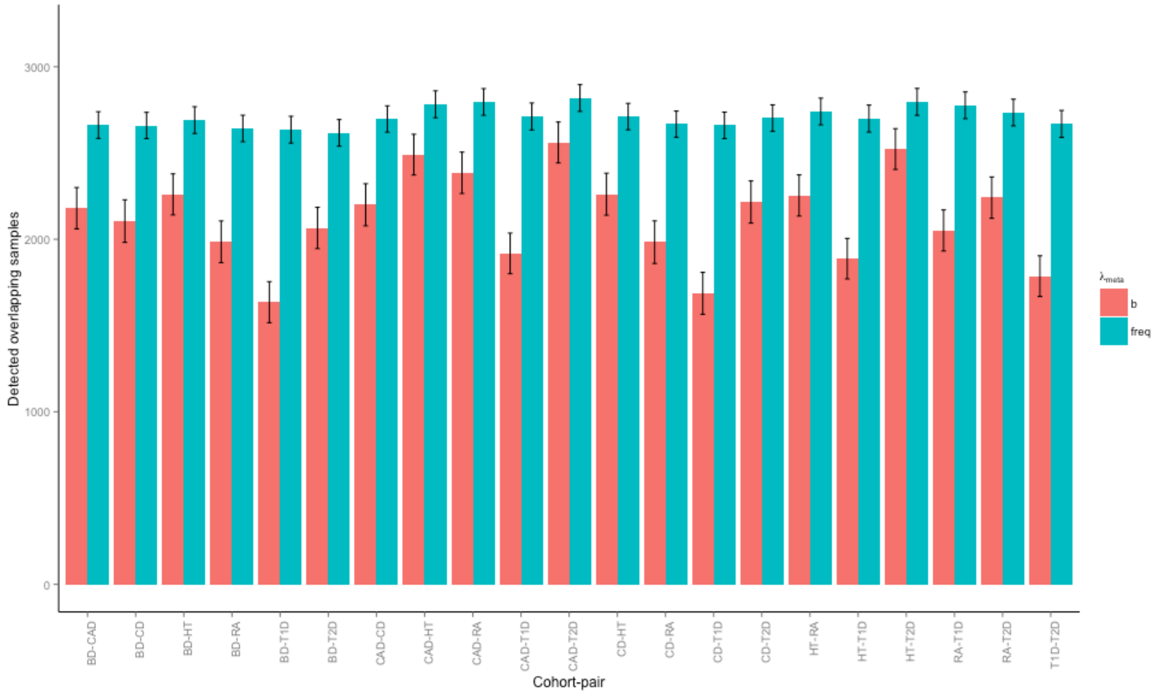

Figure S12 Workflow for PPSR regression.

Step 1: determine the number of pseudo profile scores. Given experiment-wise type I error rate = 0.01, type II error rate = 0.05 (power = 0.95). K pseudo profile scores should be generated using M markers, which guarantees the privacy of individual genotypes. **Step 2**: generate profile scores for each cohort. The meta-analysis center generates a KXM matrix for pseudo genetic effects. In total K profile scores were generated for each individual in each cohort. **Step 3**: PPSR method for detecting overlapping individuals using profile scores. For a pair of cohorts, PPS regression was conducted for each possible pair of individual for any two cohorts over the generated pseudo-profile scores. Once the regression coefficient was greater than the threshold, here *b=0.95*, the pair of individual was inferred to be having highly similar genotypes, which may indicate the individual has been included in both cohorts.

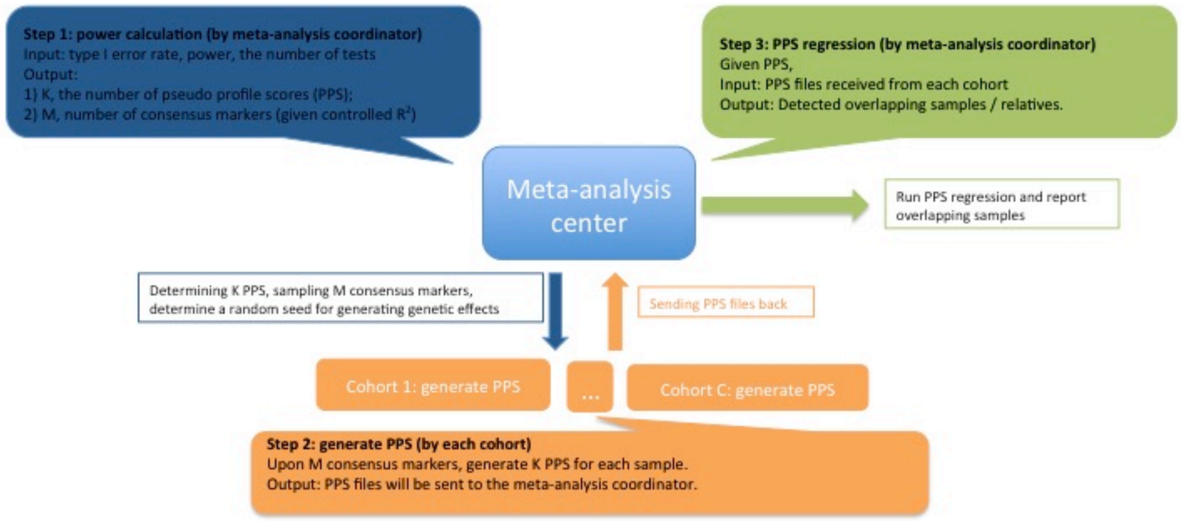

Supplement: Supplementary Figures [file ejhg2016106x1.pdf]
