# Supplementary material for: Across-cohort QC analyses of GWAS summary statistics from complex traits
Source: Eur J Hum Genet. 2016 Aug 24;25(1):137–46. doi: 10.1038/ejhg.2016.106 (PMC5159754; doi:10.1038/ejhg.2016.106)
Supplement: Supplementary Notes [file ejhg2016106x2.doc]

**Title:** Across-cohort QC analyses of GWAS summary statistics from complex traits

**Authors:** Guo-Bo Chen^1^, Sang Hong Lee^1,2^, Matthew R Robinson^1^, Maciej Trzaskowski^1^, Zhi-Xiang Zhu^3^, Thomas W Winkler^4^, Felix R Day^5^, Damien C Croteau-Chonka^6,7^, Andrew R Wood^8^, Adam E Locke^9^, Zoltán Kutalik^10-12^, Ruth J F Loos^13-15^, Timothy M Frayling^8^, Joel N Hirschhorn^16-19^, Jian Yang^1,21^, Naomi R Wray^1^, The Genetic Investigation of Anthropometric Traits (GIANT) Consortium^20^, Peter M Visscher^1,21^

**Affiliations:**

^1^ Queensland Brain Institute, The University of Queensland, Brisbane, Queensland, Australia

^2^ School of Environmental and Rural Science, The University of New England, Armidale, New South Walsh, Australia

^3^ SPLUS Game, Guangzhou, Guangdong, China

^4^ Department of Genetic Epidemiology, Institute of Epidemiology and Preventive Medicine, University of Regensburg, Regensburg, Germany

^5^ Medical Research Council (MRC) Epidemiology Unit, Institute of Metabolic Science, Addenbrooke’s Hospital, Cambridge, UK

^6^ Department of Genetics, University of North Carolina, Chapel Hill, North Carolina, USA

^7^ Channing Division of Network Medicine, Department of Medicine, Brigham and Women’s Hospital and Harvard Medical School, Boston, Massachusetts, USA

^8^ Genetics of Complex Traits, University of Exeter Medical School, University of Exeter, Exeter, UK

^9^ Department of Biostatistics and Center for Statistical Genetics, University of Michigan, Ann Arbor, Michigan, USA

^10^ Department of Medical Genetics, University of Lausanne, Lausanne, Switzerland

^11^ Institute of Social and Preventive Medicine (IUMSP), Centre Hospitalier Universitaire Vaudois (CHUV), Lausanne, Switzerland

^12^ Swiss Institute of Bioinformatics, Lausanne, Switzerland

^13^ The Charles Bronfman Institute for Personalized Medicine, Icahn School of Medicine at Mount Sinai, New York, New York, USA

^14^ The Mindich Child Health and Development Institute, Icahn School of Medicine at Mount Sinai, New York, New York, USA

^15^ The Genetics of Obesity and Related Metabolic Traits Program, Icahn School of Medicine at Mount Sinai, New York, New York, USA

^16^ Department of Genetics, Harvard Medical School, Boston, Massachusetts, USA

^17^ Program in Medical and Population Genetics, Broad Institute of MIT and Harvard, Cambridge, Massachusetts, USA

^18^ Center for Basic and Translational Obesity Research, Boston Children's Hospital, Boston, Massachusetts, USA

^19^ Division of Endocrinology, Boston Children's Hospital, Boston, Massachusetts, USA

^20^ A full list of members is available in the **Supplementary Note**

^21^ The University of Queensland Diamantina Institute, Translation Research Institute, Brisbane, Queensland, Australia

**Correspondence should be addressed to**

GBC ([chenguobo@gmail.com](mailto:chenguobo@gmail.com)) or PMV ([peter.visscher@uq.edu.au](mailto:peter.visscher@uq.edu.au))

**Table of contents**

Overview of four metrics [3](#__RefHeading___Toc325313821)

Method I:
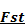
 derived genetic distance [4](#__RefHeading___Toc325313822)

Method II: Principal component analysis for cohort-level allele frequencies [7](#__RefHeading___Toc325313823)

Method III: The detection of overlapping samples with
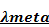
 [8](#__RefHeading___Toc325313824)

Method IV: Pseudo profile score regression (PPSR) [13](#__RefHeading___Toc325313825)

Supplementary Note I:
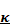
 for case-control study in
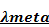
 [18](#__RefHeading___Toc325313826)

Supplementary Note II: get sampling variance for
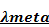
 [19](#__RefHeading___Toc325313827)

Supplementary Note III: The connection between
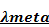
 and LD score regression [22](#__RefHeading___Toc325313828)

Supplementary Note IV: Direct correlation between test statistics [23](#__RefHeading___Toc325313829)

References [25](#__RefHeading___Toc325313830)

GIANT Authors [27](#__RefHeading___Toc325313831)

# Overview of four metrics


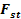
**-based inference of cohort origins (**
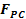
**).** For each cohort, its
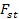
 with reference cohorts, such as CEU, YRI, and CHB, is calculated. Given those three
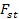
 values to the reference cohorts, the coordinate of an individual cohort can be uniquely projected into the reference equilateral that has CEU, YRI, and CHB at its corners (see **Supplementary Notes for Method I**).

**Principal component analysis for cohort-level allele frequencies (meta-PCA).** A genetic relationship matrix for cohorts can be constructed based on received allele frequencies. Principal component analysis (PCA) can be implemented on the genetic relationship matrix. The projection of the cohorts into PCA space can reveal the genetic background and relative geographical distance between cohorts (see **Supplementary Notes for Method II**).


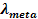
 **for detecting the proportion of overlapping samples.** In concept,
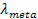
 resembles
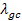
, which indicates population stratification for a GWAS^1^, but
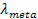
 measures the proportion of overlapping samples between a pair of cohorts. Based on reported genetic effects and their sampling variance,
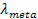
 can be constructed for a pair of cohorts and follows a chi-square distribution with 1 degree of freedom.
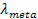
 will be close to 1 when there are no overlapping samples, smaller than 1 when there are overlapping samples, and greater than 1 when there are heterogeneity between a pair of cohorts. For GWAMA over a single trait, we assume heterogeneity is zero (see **Supplementary Notes for Method III**).

**Pseudo profile score regression for pinpointing overlapping samples/relatives.** Pseudo profile score regression (PPSR) provides a framework for pinpointing the overlapping samples/relatives between cohort without sharing genotypes. Each GWAS analyst generates pseudo profile scores (PPS) for each sample on a set of loci, which are chosen by a GWAMA central analyst. If the similarity metric of PPS for a pair of cohorts reaches a similarity threshold, say 1 for overlapping samples and 0.5 for first-degree relatives, then overlapping samples/relatives are found. PPSR can have a controlled type-I and type-II error rates in pinpointing overlapping samples, and also can reduce the comprise of privacy. PPSR is an enhanced version of Gencrypt^2^, a previous method in pinpointing overlapping samples (see **Supplementary Notes for method IV**).

# Method I:
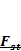
 derived genetic distance


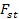
is a measure of genetic differentiation between populations. It is usually estimated using individual-level genotype data from multiple samples in two or more populations^3^. Here, we calculate
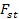
 using summary data on allele frequencies, which implicitly assumes Hardy-Weinberg equilibrium genotype frequencies within populations. We use summary statistic calculated
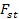
 as a metric for quality control for each cohort. If the allele frequencies reported for a cohort depart genome-wide from its expectation based on known ancestry due to technical artifacts, then we may observe an unexpected
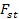
 value when comparing to a reference panel of know ancestry.

We calculate
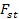
 between each cohort and a reference panel, choosing the appropriate reference sample depending on the purpose of the analysis. For the inference of global-level diversity, we chose YRI, CHB, and CEU as the reference panels. For the inference of within-Europe diversity, we chose CEU, FIN, and TSI as the reference panels. As the different allele frequencies across three samples reflected the real diversity among these reference panels, we did not apply any exclusion criteria on the reference allele frequency. Nevertheless, as GIANT height GWAS samples were imputed to the HapMap panel, the majority of SNPs matched to the 1KG reference samples comprised common SNPs. After ranking the calculated
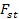
 in ascending order for all matched SNPs, we sampled 30,000
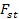
 evenly along the ordered
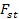
. These 30,000 markers are quasi-independent and evenly distributed across the genomes. The mean of the 30,000
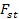
 was employed to represent the
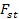
 measure between a cohort and a reference panel. The sampled 30,000 markers may differ from one pair of cohorts to another pair, but as tested resample 30,000 markers caused ignorable changes of the mean of
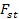
. Another reason we chose 30,000 markers is that there are around 30,000 quasi-independent markers for GWAS data as observed in empirical data and expected from theory^4,5^.

In this study,
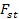
 is calculated from the allele frequencies estimated from cohorts, provided as summary statistics.
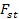
 is treated as a data statistic for measuring allele frequency differentiation. In general the interpretation of
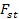
 can vary with context^6^.


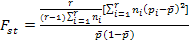
 **(Equation 1)**

with
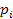
 the estimated reference allele frequency in population
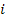
 from a sample of
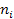
 alleles,
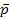
 is the weighted average frequency in the entire sample, and *r* is the number of populations. Here, we only compared each cohort to the 1KG reference panel, so
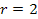
 and the equation becomes


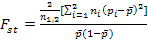
 **(Equation 2)**

in which
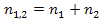
, and
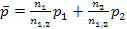
 is the mean allele frequency. Alternative estimators for
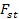
 are possible, and a comprehensive comparison of different
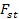
 estimators was recently reported^7^.

If the two cohorts are not that different in terms of their allele frequencies, for example, the cohorts from European nations,
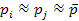
,


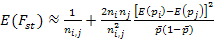
 **(Equation 3)**

At the right side of the equation, the first term represents the sampling variance for allele frequency for a pair of cohorts, and the second term represents the allele frequency difference due to divergence from a common ancestor. The estimated
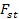
 is influenced by sample size, and
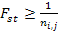
, which is the sampling variance of
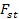
 for a pair of cohorts^3^. As each 1KG reference population has a sample size around 100, there is no disproportionate impact of sample size in calculating
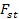
.


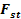
 **Cartographer algorithm.** The purpose of using the
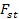
 Cartographer algorithm is to find the coordinates of a cohort given its
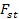
 to the reference populations. The algorithm can be expressed in Cartesian geometry. Given three reference populations, a target cohort has three
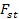
 measures,
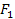
,
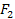
, and
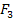
, respectively. Given a Cartesian coordinate system, the coordinate for these three reference populations are
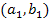
,
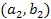
, and
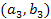
, respectively. The algorithm tries to find the coordinates
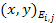
 on each the edge
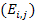
 that connects reference populations
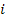
 and
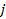


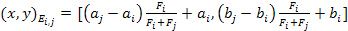
 **(Equation 4)**

The coordinates of the gravity of triangle,
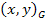
, that connects
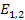
,
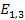
, and
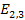
 are


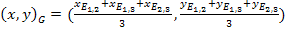
 **(Equation 5)**

**Inference of cohort origins at the global level.** To assess genetic background, for each cohort we calculated its
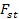
values using CEU, CHB, and YRI as the reference panel, respectively. We denote these three
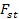
 values as
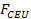
,
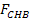
, and
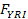
. These values reflect genetic distances between a cohort and the reference panels - the greater the value the further the genetic distance. We developed an algorithm called
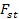
 cartographer, which can map a cohort to global genetic variation as previously observed using individual level data from principal component analysis^8^. The steps in the algorithm are as follows (**Fig. S1**):

Create the coordinates for the reference samples. Without loss of generality, these three reference populations form an equilateral triangle, and we set the length of each edge to unity. For example, the coordinates CEU, CHB, and YRI are (
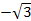
, 1), (
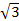
, 1), and (0, -2), respectively, and connecting the coordinates of the three reference populations formed an equilateral triangle – the reference space**.** The gravity of this equilateral triangle is the origin of the Cartesian space. The choice for the coordinates for the reference population is arbitrary.

**Step 1** **Create a cohort triangle using Equation 4.** Finding a point the distances of that to both ends, which represent two populations, is proportional to the ratio of the
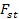
 values of the cohort to these two reference populations. Similarly, find the points on the other two edges. For example, Finland Twin Cohort (FTC) had
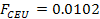
,
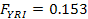
, and
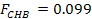
. On the CEU-YRI edge, a point split the length to 0.0102:0.153, was
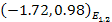
; On the CEU-CHB edge into 0.0102:0.099, was
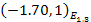
; and on the YRI-CHB edge into 0.153:0.099, was
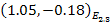
. Connecting the three coordinates created a “FTC” triangle inside the reference triangle.

**Step 2** **Find the gravity of the cohort triangle using Equation 5.** The gravity of the “FTC” triangle had its coordinates of
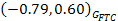
, which is inferred as the geographic coordinates for FTC in
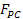
 space. It had relative distances of 1.03, 2.55, and 2.72 to CEU, CHB, and YRI, respectively. The shorter the distance, the closer the genetic background is.

**Step 3** Repeat Steps 1, and 2 until the gravity of each cohort is found.

Plots of the coordinates for each cohort will show the relative distance of each cohort to the reference samples. If a cohort has equal distances to three reference populations, its gravity will be close to the origin of the reference triangle.

# Method II: Principal component analysis for cohort-level allele frequencies

PCA has been widely used in genetics^9^ and was recently proposed for controlling population stratification for GWAS^10,11^. We provide a new method that uses cohort-level allele frequencies, often provided as summary statistics in meta-analysis. We call the new method as meta-PCA.

Meta-PCA is based on a
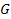
 matrix, which includes
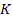
 reference populations and
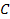
 cohorts with data on
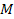
 markers. In
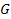
, the
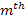
 column represents the reported reference allele frequencies for the
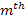
 marker for (K+C) cohorts. The kernel correlation matrix for PCA is constructed on
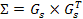
, in which
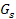
 is the standardization for
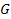
 for each locus (on each column of
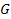
). Compared with individual-level data PCA, in the context of meta-PCA each cohort can be viewed as an individual in the conventional sense. Given the
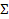
 matrix, the implementation is the same as individual-data PCA.

There are insightful reports on the genetic interpretation for PCA^10,12–14^. The interpretation of meta-PCA could be approached by
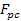
 as described in the previous section.

# Method III: The detection of overlapping samples with
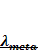


**Inference of cohort origins at the within-Europe level.** To assess genetic background, for each cohort we calculated its
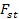
values using CEU, FIN, and TSI as the reference panel, with coordinates (, 1), (, 1), and (0, -2), respectively. For FTC, it had values of 0.0102, 0.0052, and 0.0157, to CEU, FIN, and TSI, respectively. Using the Cartographer algorithm, the gravity of the FTC triangle had its coordinates of . It had relative distances of 2.10, 1.59, and 2.42, to CEU, FIN, and TSI, respectively.

**Genealogical subspace.** Furthermore, we partition the space into three subspaces. For example, given coordinates of (, 1), (, 1), and (0, -2), for CEU, FIN, and TSI, respectively, connecting the origin and the coordinates for any two reference populations created a subspace, which is defined as a genealogical subspace. We had three genealogical subspaces: CEU-FIN genealogical subspace, CEU-TSI genealogical subspace, and FIN-TSI genealogical subspace, respectively. If a cohort is located inside a subspace, it indicates that this cohort may be derived from these two reference populations that creates the genealogical subspace.

For European cohorts, the coordinates calculated from Cartographer algorithm mirror the origins of geographic locations of the cohorts, similar, but less refined, to what has been observed in previous studies using individual level data for European samples^15,16^.

**Effective number of overlapping samples** **.** If a pair of cohorts has overlapping samples, it leads to a correlation of the estimated genetic effects for each locus. In the recent literature, two kinds of correlation due to overlapping samples were introduced. The first one was defined by directly calculating correlation between all estimated test statistics, , in which is a vector of matched loci between two cohorts ^17,18^. The second one was defined on the correlation for single locus given overlapping samples, as introduced by Lin and Sullivan^19^. We used the second definition, and then extended the correlation due to any relatives, a generalization of Lin and Sullivan.

For a pair of cohorts of sample sizes and , for matched loci which have GWAS summary statistics, for example additive effects and their standard errors. For the locus, estimated association effect sizes are and with sampling variance and , respectively. is assumed to be drawn from a normal distribution , and . In cohort 1, is proportion of samples with a -degree relatives in cohort 2 with the number of relatives of kth degree relatives shared between the samples; the phenotypic variance is assumed to be the same across the cohorts for a quantitative trait. For a locus, the genetic effect is estimated by linear regression in cohort 1 (the index for the locus is dropped for convenience). If the sampling variance of a locus is assumed to be the same for any subset of samples

The standard error of is , in which is the proportion of phenotypic variance explained by the locus and is the phenotypic variance of the trait. The sampling variance for . This decomposition of the genetic effect can be applied to cohort 2. Consequently, the covariance between and for the locus is

in which is the covariance between the genetic effects estimated in two cohorts due to the -degree relatives. is the phenotypic correlation for the -degree relatives, and is the genetic relatedness for the -degree relatives. is the coefficient of identity for descent. For duplicated samples, , in which is the heritability, and , the environmental correlation to be close 1 for overlapping samples; for other relatives (), .

**Correlation between the estimated genetic effects.** The covariance can be generalized as . After adjustment by the sampling variance, the correlation between and is

**(Equation 6)**

in which , is the effective number of overlapping samples averaged over all relative pairs that are across the two cohorts. As the variance explained by each locus is small, and after further weighted by , the contribution from overlapping relative is small. When ignoring the first and higher degree relatives equals the contribution from overlapping samples. This is consistent with the results from Lin and Sullivan^19^, who considered overlapping samples only. So, the correlation at any single locus is largely determined by the overlapping samples () for summary statistics.

**(Equation 7)**

So, in the text hereafter, indicates overlapping samples only, otherwise specified.

**Correlation for case-control studies.** The theory above is based on a quantitative trait, but it holds approximately true for case-control studies if a locus is from the null distribution of no association with the disease. Given overlapping controls and overlapping cases, for a locus associated with disease its correlation of the regression coefficient is as indicated by Lin and Sullivan^19^, in which is the ratio between cases and controls in the cohort. When it is balanced case-control design – , resembles the correlation for quantitative traits. However, it should be noticed that for case control data, is confounded with the number of overlapping cases and controls.

**Theory for** **.** For the summary statistics between a pair of cohorts for the locus, we can construct a statistic

**(Equation 8)**

in which is the correlation between and .

**(Equation 9)**

in which , , and , as defined in Equation 7, is the correlation for this locus due to overlapping samples between this pair of cohorts. Of note, is same for each locus regardless of a null locus or a locus associated to genetic effects. For convenience, the subscript was dropped in the text hereafter.

Under the null hypothesis of no heterogeneity () and no correlation (), , a standard 1-degree-of-freedom chi-square distribution. , in which is the effective number of overlapping samples. Of note, since the majority of markers are likely sampled from the null distribution or have very small effect sizes, we can approximate and , and therefore for most marker pairs between a pair of cohorts. For the marker that is in linkage disequilibrium with causal variants, , in which is the number of causal variants in linkage disequilibrium with the marker for cohort 1, is the causal variants in linkage disequilibrium with the marker, and is the LD correlation between the marker and the causal variant^20^. Similarly for . If the cohorts are from the same ethnicity, the difference in the LD correlation can be ignored, for example for samples from cohorts with European ancestry. So, under a polygenic model is expected to be zero, or close to zero.

The statistic is calculated for each matched SNP between a pair of cohorts. After ordering all values, we evenly sample 30,000 independent markers from the order statistic of all values. Each pair of cohorts may sample values based on 30,000 markers different from another pair of cohorts.

**(Equation 10)**

in which . Under the null hypothesis of no heterogeneity and overlapping samples , plotting the ordered against its corresponding quartiles from , will be along the diagonal, leading to . Heterogeneity between two cohorts, equivalent to a “negative” number of overlapping samples, will drive , and overlapping samples will make . The distribution of can be assessed via the beta distribution, and follows asymptotically a normal distribution given 30,000 independent markers.

**Factors that influence** **.** A number of factors will influence the . 1) Sample overlap, including close relatives across cohorts, reduces the value of (**Fig. S2**) Conservative modeling, such as inclusion of covariates in the association model that are genetically correlated with the phenotype or the ‘genomic control’ approach (adjusting the sampling variance with , ), will inflate the sampling variance, and deflate . 3) Genetic heterogeneity, which can be caused by differences in genetic architecture or methodological difference, will inflate . 4) As characterized by Equation 10, the lower bound (cohort 2 is completed included in cohort 1, given ) of is , upon the ratio of the samples sizes of the two cohorts.

**Estimating overlapping samples.** As shown in Equation 10, is a linear function of , hence the statistical power to detect overlapping samples is equivalent to asking how departs from the null distribution. Assuming , the overlapping samples can be estimated as , and given 30,000 independent markers. Hence, using summary statistics only the proportion of overlapping samples can be estimated for quantitative traits. Given the type I error rate of 0.05 , the statistical power for detecting overlapping samples between two cohorts is , in which represents the accumulation power function of a normal distribution with the mean of and standard deviation of . The statistical power is determined by , the threshold for significance, , the real overlapping samples, and , the standard deviation of the null hypothesis that there is no overlapping samples. Without loss of generality, given . The 95% confidence interval is . The statistical power is maximized when , i.e. when a pair of cohorts has the same sample size.

For case-control studies, as , the estimate cannot distinguish between overlapping cases and overlapping controls; when and (balanced case-control design for both cohorts), , indicating the overall overlapping samples between two cohorts, summed across cases and controls. If we know that only controls (cases) were shared between two cohorts, then (), so then an estimate of indicates the number of overlapping controls (cases). Therefore, quantifying overlapping samples for case-control studies is more difficult than that for quantitative traits.

# Method IV: Pseudo profile score regression (PPSR)

**PPSR** resembles the previously proposed Gencrypt method^2^, but PPSR is more powerful in detecting various degree of relatives and more robust to missing data and imputation errors. For each individual, the PPS can be generated as below

**(Equation 11)**

in which is the PPS for the individual, is a score matrix, and is vector for the genotypes for the chosen loci.

In detail,

in which is the profile score for the individual, is the additive effect at the locus ( from 1 to ) for the profile score, and is the standardized genotype at the locus for the individual. Each , the pseudo genetic effect, follows a standard normal distribution ; each pseudo genetic effect is independent to another. For each PPS, , in which is the column for the matrix, and on average each locus explains of the variation. For an individual a pair of PPS, say and , has .

Each PPS can be seen as a trait with because it does not have any sampling variance. For a pair of individuals, individual and individual , when both and have been standardized, their covariance for the PPS , in which is the relatedness scores in terms of identity by state^21^. Depending on the relatedness between a pair of individuals, for monozygous twins or to a duplicated sample, for first-degree relatives such as parent and offspring or full sibs. In general, for -degree of relatives, .

The theory presented above provides a theoretical basis for detecting overlapping samples using PPS other than sharing individual level genotypes. Assuming that each individual has independent PPS ( having elements), for individual and , we can regress on ,

**(Equation 12)**

in which is the grand mean, is the regression coefficient, and is the residual. . if individual is not correlated with individual , for first-degree relatives, and if individual and are genetically same, say an overlapping sample or the homozygous twins. The sampling variance of is . Under the null distribution for no related or overlapping samples, . The residual accounts the discordant genotypes, including missing genotypes and genotyping or imputation errors. For current GWAS data, after quality control, the discordant rate is often smaller than 1%.

If now we have cohorts for which the individual genotypes of which cannot be disclosed to the central analysis hub, overlap between cohorts can be identified if PPS are supplied. By regressing their PPS to each other the overlapping individuals could be detected if . Assuming there are samples in each cohort, a total of regressions need to be carried out as defined in Equation 12. If we want to control the experiment-wise type I error rate under the null hypothesis and type II error rate (with power) for , the required number of pseudo profile scores for each individual is

**(Equation 13)**

in which and are scores under the given *p*-values at the subscripts. To accommodate technical errors, such as missing genotypes and genotype error, a cutoff of 0.95 for is adopted for detection of overlapping samples, and for detecting first-degree relative.

The standardization of genotypes can either use the allele frequency from each cohort, or from a reference sample. Throughout the study, we used the allele frequency calculated from WTCCC bipolar disorder cohort as the reference, and using it as an approximation to standardize genotypes for all cohorts in comparison.

**Workflow for PPSR.** Given the statistical method for detecting overlapping samples as described above, the whole workflow for detecting can be split into three steps (**Fig. S12**).

**In step 1, the required type I and type II error rates are defined and from that the required number of pseudo profiles to be generated.** The GWAMA central analyst selects consensus SNP markers across cohorts, and determines additive effects matrix that will be used to generate pseudo profile scores for each cohort. In order to avoid strand issues, the loci having palindromic loci (A/T alleles or G/C alleles) are excluded.

**In step 2, each cohort generates PPSR for each individual with the set of consensus markers and the marker weights received from the GWAMA coordinator.** After generate the PPS, they send them back to the coordinator. This will be a file that contains N rows and K columns with pseudo-profile scores.

**In step 3, the coordinator runs PPSR for each sample in a cohort on each PPS generated for another cohort.** The final product of running PPSR is to generate a matrix for a pair of cohorts, which have and samples respectively. For each pair of individuals in comparison, we take the one from cohort as the response variable and from cohort as the predictor variable in PPSR. In principle, swapping the response variable and the predictor variable do not affect the performance of PPSR. Each entry, the regression coefficient of PPSR, in the matrix represents genetic similarity for these pair of individuals in comparison. Once the regression coefficients are above the threshold, it indicates there are samples duplicated. The central analyst can then request each cohort that is implicated in containing samples that are also in other cohorts to drop those samples, without revealing where the duplication occurred.

**Privacy issues when using PPSR.** As the exchange of the PPS is within a meta-analysis facility, it is not as vulnerable as that of releasing the GWAS summary to the public domain as discussed in previous studies^22–24^. However, as PPS are generated from genotypes, it is worth to consider whether the PPS will reveal individual genotype information, or can be decoded from PPS. As a demonstration for the principle-of-proof, we consider to reverse Equation 11 to estimate genotypes. We consider the case where the additive effect matrix in Equation 11 is known, otherwise it is nearly impossible to recover genotype information. Given the workflow of PPSR, the analysts who coordinate the meta-analysis know the additive effect matrix, in Equation 11, and receive PPS from each cohort have the information to decode genotypes that are employed to generate PPS.

After reversing Equation 11, using the standard regression method, the genotype in each locus can be estimated as

**(Equation 14)**

In detail,

in which is the column in the additive effects matrix in Equation 11. Although , which is an unbiased estimate of the genotype, its sampling variance is . The sampling variance can be further written as because and is denoted as . The greater the ratio between and , the larger the sampling variance, and consequently the lower probability to construct the real genotype.

Without loss of generality, the accuracy of the estimated , a continuous variable, and , a discrete variable with values of 2, 1, and 0, can be measure using the squared correlation ()^25^,

**(Equation 15)**

in which and are:

, , and if is the reference allele, and , , and are weighted frequency given the distribution of . .

When the reference allele frequency follows a uniform distribution between , assuming that the loci follow Hardy-Weinberg proportions, , , and , in which follows a uniform distribution between and and .

and , , and .

If the reference allele frequency follows a uniform distribution between (0, 0.5), .

Given loci with MAF of 0.5, the expected frequencies for , , and are , , , and ., and . Plugging them in to the Equation 13 leads to .

Equation 13 can be rewritten as , in which if MAF is 0.5, and if MAF in nearly from a uniform distribution. From Equation 13, it is easy to calculate the ratio between the number of markers and the number of PPS given a controlled ,

**(Equation 16)**

For uniform distribution of MAF, if is set as the threshold, ; if , , and if , . In general, the higher the ratio between and , the less information can be inferred. We suggest may be sufficient.

The speed of PPSR depends on , the sample sizes for a pair of cohorts, and the number of PPS for each cohort; for the WTCCC data there are 21 cohort-pair comparisons, and each pair took about 20 minutes, on a computer with a 2.3 GHz CPU, given about comparisons. The average sample size of GIANT is about 1,500, and takes about 2 minutes for each pair of cohorts. The two largest datasets are deCODE with 26,790 samples and WGHS with 23,100 samples, and PPSR to detect overlapping samples takes about 8.5 hours. As each pair of individuals is computationally an independent unit, analysis jobs can be parallelized on a cluster. Therefore, even for meta-analyses involving many large cohorts, the computation time is not a limiting factor.

# Supplementary Note I: for case-control study in

For case-control data, we use logarithm transformation for odds ratio, and its sampling variance for is , in which is the number in a contingency table for case-control data.

|  | A | a |  |
| --- | --- | --- | --- |
| Cases |  |  |  |
| Controls |  |  |  |
|  |  |  |  |

For a null locus, and .

. For a null locus, and ;

, in which and ;

. So, regardless of quantitative traits or case-control studies, is same.

# Supplementary Note II: get sampling variance for

Derivation of the sampling variance of is bypassed through beta distribution. Under the null distribution, the median of the sampled independent has p-value of 0.5 in . Furthermore, as order statistic, this p-value follows , which is a beta function with the shape parameters and the scale parameter . It has mean , and standard deviation . In this study, by default, we take because GWAS data often has around 30,000 quasi-independent markers^4,5^. Given , . And the 95% confidence interval for p-value is (0.494, 0.505), with the corresponding chisq values of 0.443 and 0.467. The 95% confidence interval for is , or . A score test can be constructed, . Similar method could be used to estimate sampling variance for conventional .

**Supplementary Note III: constructing**  **on allele frequency from summary statistics**

Summary statistics observed as listed in the table below

| SNP | Sample size | Effect (S.E.) |  | RAF (S.E.) |
| --- | --- | --- | --- | --- |
| Cohort 1 | | | | |
| Marker 1 |  |  |  |  |
| Marker 2 |  |  |  |  |
|  |  |  |  |  |
| Marker |  |  |  |  |
| Cohort 2 | | | | |
| Marker 1 |  |  |  |  |
| Marker 2 |  |  |  |  |
|  |  |  |  |  |
| Marker |  |  |  |  |

Assuming we have two cohorts, which have and samples in each, respectively. Within each cohorts, the samples are unrelated to each other. These two samples have overlapping samples. For the locus, the reference allele frequency can be calculated as below (the subscript for locus is dropped for convenience)

whence is the frequency of the reference allele for the cohort 1, is the frequency of the reference allele for the overlapping samples, for the rest of the samples; is the proportion of the overlapping samples in cohort 1, and for the rest. The allele frequency for the other allele is .

and

reflects the variation due to genetic drift in two subsamples. When the cohort is homogeneous in allele frequency , ; . Similarly for cohort 2, . Of note, .

The covariance between and is

The correlation between and is

When there is no heterogeneity, , between cohorts in allele frequency .

in which , indicating allele frequency difference/population stratification, and , which is a known constant. Define . If there is no population stratification, .

Calculate for each pair of markers, and make order statistics for all . Evenly sample elements. Under the null hypothesis of no overlapping samples and population stratification between two cohorts the sampled sequence should follow a chi-squared distribution of 1 degree of freedom. And .

# Supplementary Note III: The connection between and LD score regression

Recently LD regression has been proposed^26^. It uses the linkage disequilibrium scores estimated from a reference population as instrument variable. can be transformed into the form of LD regression as below.

If we construct . And .

, in which , , and . follows .

If regression on LD scores, it will be , in which , is the LD score, and a function of heterogeneity.

When there is no overlapping samples, ; otherwise, . is the regression coefficient of heterogeneity.

# Supplementary Note IV: Direct correlation between test statistics

| **Notations** | **Definitions** |
| --- | --- |
|  | Phenotypic correlation for a pair of traits for two cohorts. |
|  | , is the correlation due to overlapping samples. is the number of overlapping samples, and are samples sizes for two cohorts. |
|  | Genetic correlation. for the same trait. |
|  | The correlation estimated from summary statistics, and |
|  | The correlation component due to overlapping samples for the estimated correlation from summary statistics. |
|  | The correlation component due to genetic component for the estimated correlation from summary statistics. The upper bound for . |
|  | , the proportion of the correlation due to overlapping samples. |

The correction can also be estimated from z-scores for a pair of cohorts. Similarly, depending on whether the locus is causal or not, scores can be split into two distributions. Now, for a pair of cohorts

For a mixture distribution, its variance is

and the covariance is

in which and . is the number of QTLs for each cohort, and is the number of loci tested. For the same trait, . For the case above, as ,

We have

in which is the overlapping samples between two cohorts, and and are the sample sizes for two cohorts respectively.

The correlation between two z-score sequences is

As the overlapping samples are confounded with genetic architecture, it can be decomposed into two components

in which is the genetic correlation between the two sets of z score sequences, and are heritabilites for two cohorts respectively.

**Remark 1:** The correlation will be unit if the sample sizes of both cohorts go infinite as long as .

**Remark 2:** When , . It shows some connection to the prediction theory^25,27^, the prediction accuracy is .

The correlation part due to overlapping sample is

**Remark 3:** the estimated correlation due to overlapping samples is not greater than . When heritability is zero, and the traits are same, .

**Remark 4:**  increases with the number of markers, and has its upper bound .

The correlation can be split into two components: due to heritability and due to overlapping samples. The proportion due to the overlapping samples is

in which is the phenotypic correlation between two cohorts. For the same trait, , and too.

# References

1 Devlin B, Roeder K. Genomic control for association studies. *Biometrics* 1999; **55**: 997–1004.

2 Turchin MC, Hirschhorn JN. Gencrypt: one-way cryptographic hashes to detect overlapping individuals across samples. *Bioinformatics* 2012; **28**: 886–8.

3 Weir BS. *Genetic data analysis*. 2nd ed. Sinauer Associates, Inc.: Sunderland, MA, USA, 1996.

4 Vinkhuyzen AAE, Wray NR, Yang J, Goddard ME, Visscher PM. Estimation and Partition of Heritability in Human Populations Using Whole-Genome Analysis Methods. *Annu Rev Genet* 2013; **47**: 75–95.

5 Chen G-B. Estimating heritability of complex traits from genome-wide association studies using IBS-based Haseman-Elston regression. *Front Genet* 2014; **5**: 107.

6 Weir BS, Cockerham CC. Estimating F-Statistics for the Analysis of Population Structure. *Evolution (N Y)* 1984; **38**: 1358–1370.

7 Bhatia G, Patterson N, Sankararaman S, Price AL. Estimating and interpreting FST: the impact of rare variants. *Genome Res* 2013; **23**: 1514–21.

8 Novembre J, Johnson T, Bryc K *et al.* Genes mirror geography within Europe. *Nature* 2008; **456**: 98–101.

9 Cavalli-Sforza LL, Menozzi P, Piazza A. *The History and Geography of Human Genes*. Princeton University Press, 1996.

10 Patterson N, Price AL, Reich D. Population structure and eigenanalysis. *PLoS Genet* 2006; **2**: e190.

11 Price AL, Patterson NJ, Plenge RM, Weinblatt ME, Shadick N a, Reich D. Principal components analysis corrects for stratification in genome-wide association studies. *Nat Genet* 2006; **38**: 904–9.

12 McVean G. A genealogical interpretation of principal components analysis. *PLoS Genet* 2009; **5**: e1000686.

13 Bryc K, Bryc W, Silverstein JW. Separation of the largest eigenvalues in eigenanalysis of genotype data from discrete subpopulations. *Theor Popul Biol* 2013; **89**: 34–43.

14 Chen G-B, Lee SH, Zhu Z-X, Benyamin B, Robinson MR. EigenGWAS: finding loci under selection through genome-wide association studies of eigenvectors in structured populations. *bioRxiv* 2015; : dx.doi.org/10.1101/023457.

15 Novembre J, Stephens M. Interpreting principal component analyses of spatial population genetic variation. *Nat Genet* 2008; **40**: 646–9.

16 Mcevoy BP, Montgomery GW, Mcrae AF *et al.* Geographical structure and differential natural selection among North European populations. *Genome Res* 2009; **19**: 804–814.

17 Bolormaa S, Pryce JE, Reverter A *et al.* A multi-trait, meta-analysis for detecting pleiotropic polymorphisms for stature, fatness and reproduction in beef cattle. *PLoS Genet* 2014; **10**: e1004198.

18 Zhu X, Feng T, Tayo BO *et al.* Meta-analysis of Correlated Traits via Summary Statistics from GWASs with an Application in Hypertension. *Am J Hum Genet* 2015; **96**: 21–36.

19 Lin D-Y, Sullivan PF. Meta-analysis of genome-wide association studies with overlapping subjects. *Am J Hum Genet* 2009; **85**: 862–72.

20 Yang J, Weedon MN, Purcell S *et al.* Genomic inflation factors under polygenic inheritance. *Eur J Hum Genet* 2011; **19**: 807–12.

21 Powell JE, Visscher PM, Goddard ME. Reconciling the analysis of IBD and IBS in complex trait studies. *Nat Rev Genet* 2010; **11**: 800–5.

22 Homer N, Szelinger S, Redman M *et al.* Resolving individuals contributing trace amounts of DNA to highly complex mixtures using high-density SNP genotyping microarrays. *PLoS Genet* 2008; **4**: e1000167.

23 Visscher PM, Hill WG. The limits of individual identification from sample allele frequencies: theory and statistical analysis. *PLoS Genet* 2009; **5**: e1000628.

24 Sankararaman S, Obozinski G, Jordan MI, Halperin E. Genomic privacy and limits of individual detection in a pool. *Nat Genet* 2009; **41**: 965–7.

25 Dudbridge F. Power and predictive accuracy of polygenic risk scores. *PLoS Genet* 2013; **9**: e1003348.

26 Bulik-Sullivan BK, Loh P-R, Finucane HK *et al.* LD Score regression distinguishes confounding from polygenicity in genome-wide association studies. *Nat Genet* 2015; **47**: 291–295.

27 Daetwyler HD, Villanueva B, Woolliams J a. Accuracy of predicting the genetic risk of disease using a genome-wide approach. *PLoS One* 2008; **3**: e3395.

# GIANT Authors

Adam E. Locke^1*^, Bratati Kahali^2*^, Sonja I. Berndt^3*^, Anne E. Justice^4*^, Tune H. Pers^5,6,7,8*^, Felix R. Day^9^, Corey Powell^2^, Sailaja Vedantam^5,6^, Martin L. Buchkovich^10^, Jian Yang^11,12^, Damien C. Croteau-Chonka^10,13^, Tonu Esko^5,6,7,14^, Tove Fall^15,16,17^, Teresa Ferreira^18^, Stefan Gustafsson^16,17^, Zoltán Kutalik^19,20,21^, Jian’an Luan^9^, Reedik Mägi^14,18^, Joshua C. Randall^18,22^, Thomas W. Winkler^23^, Andrew R. Wood^24^, Tsegaselassie Workalemahu^25^, Jessica D. Faul^26^, Jennifer A. Smith^27^, Jing Hua Zhao^9^, Wei Zhao^27^, Jin Chen^28^, Rudolf Fehrmann^29^, Åsa K. Hedman^16,17,18^, Juha Karjalainen^29^, Ellen M. Schmidt^30^, Devin Absher^31^, Najaf Amin^32^, Denise Anderson^33^, Marian Beekman^34,35^, Jennifer L. Bolton^36^, Jennifer L. Bragg-Gresham^1,37^, Steven Buyske^38,39^, Ayse Demirkan^32,40^, Guohong Deng^41,42,43^, Georg B. Ehret^44,45^, Bjarke Feenstra^46^, Mary F. Feitosa^47^, Krista Fischer^14^, Anuj Goel^18,48^, Jian Gong^49^, Anne U. Jackson^1^, Stavroula Kanoni^50^, Marcus E. Kleber^51,52^, Kati Kristiansson^53^, Unhee Lim^54^, Vaneet Lotay^55^, Massimo Mangino^56^, Irene Mateo Leach^57^, Carolina Medina-Gomez^58,59,60^, Sarah E. Medland^61^, Michael A. Nalls^62^, Cameron D. Palmer^5,6^, Dorota Pasko^24^, Sonali Pechlivanis^63^, Marjolein J. Peters^58,60^, Inga Prokopenko^18,64,65^, Dmitry Shungin^66,67,68^, Alena Stančáková^69^, Rona J. Strawbridge^70^, Yun Ju Sung^71^, Toshiko Tanaka^72^, Alexander Teumer^73^, Stella Trompet^74,75^, Sander W. van der Laan^76^, Jessica van Setten^77^, Jana V. Van Vliet-Ostaptchouk^78^, Zhaoming Wang^3,79^, Loïc Yengo^80,81,82^, Weihua Zhang^41,83^, Aaron Isaacs^32,84^, Eva Albrecht^85^, Johan Ärnlöv^16,17,86^, Gillian M. Arscott^87^, Antony P. Attwood^88,89^, Stefania Bandinelli^90^, Amy Barrett^64^, Isabelita N. Bas^91^, Claire Bellis^92,93^, Amanda J. Bennett^64^, Christian Berne^94^, Roza Blagieva^95^, Matthias Blüher^96,97^, Stefan Böhringer^34,98^, Lori L. Bonnycastle^99^, Yvonne Böttcher^96^, Heather A. Boyd^46^, Marcel Bruinenberg^100^, Ida H. Caspersen^101^, Yii-Der I. Chen^102,103^, Robert Clarke^104^, E. Warwick Daw^47^, Anton J. M. de Craen^75^, Graciela Delgado^51^, Maria Dimitriou^105^, Alex S. F. Doney^106^, Niina Eklund^53,107^, Karol Estrada^6,60,108^, Elodie Eury^80,81,82^, Lasse Folkersen^70^, Ross M. Fraser^36^, Melissa E. Garcia^109^, Frank Geller^46^, Vilmantas Giedraitis^110^, Bruna Gigante^111^, Alan S. Go^112^, Alain Golay^113^, Alison H. Goodall^114,115^, Scott D. Gordon^61^, Mathias Gorski^23,116^, Hans-Jörgen Grabe^117,118^, Harald Grallert^85,119,120^, Tanja B. Grammer^51^, Jürgen Gräßler^121^, Henrik Grönberg^15^, Christopher J. Groves^64^, Gaëlle Gusto^122^, Jeffrey Haessler^49^, Per Hall^15^, Toomas Haller^14^, Goran Hallmans^123^, Catharina A. Hartman^124^, Maija Hassinen^125^, Caroline Hayward^126^, Nancy L. Heard-Costa^127,128^, Quinta Helmer^34,98,129^, Christian Hengstenberg^130,131^, Oddgeir Holmen^132^, Jouke-Jan Hottenga^133^, Alan L. James^134,135^, Janina M. Jeff^55^, Åsa Johansson^136^, Jennifer Jolley^88,89^, Thorhildur Juliusdottir^18^, Leena Kinnunen^53^, Wolfgang Koenig^52^, Markku Koskenvuo^137^, Wolfgang Kratzer^138^, Jaana Laitinen^139^, Claudia Lamina^140^, Karin Leander^112^, Nanette R. Lee^91^, Peter Lichtner^141^, Lars Lind^142^, Jaana Lindström^53^, Ken Sin Lo^143^, Stéphane Lobbens^80,81,82^, Roberto Lorbeer^144^, Yingchang Lu^55,145^, François Mach^45^, Patrik K. E. Magnusson^15^, Anubha Mahajan^18^, Wendy L. McArdle^146^, Stela McLachlan^36^, Cristina Menni^56^, Sigrun Merger^95^, Evelin Mihailov^14,147^, Lili Milani^14^, Alireza Moayyeri^56,148^, Keri L. Monda^4,149^, Mario A Morken^99^, Antonella Mulas^150^, Gabriele Müller^151^, Martina Müller-Nurasyid^85,130,152,153^, Arthur W. Musk^154^, Ramaiah Nagaraja^155^, Markus M Nöthen^156,157^, Ilja M. Nolte^158^, Stefan Pilz^159,160^, Nigel W. Rayner^18,22,64^, Frida Renstrom^66^, Rainer Rettig^161^, Janina S. Ried^85^, Stephan Ripke^108,162^, Neil R. Robertson^18,64^, Lynda M. Rose^163^, Serena Sanna^150^, Hubert Scharnagl^164^, Salome Scholtens^100^, Fredrick R. Schumacher^165^, William R. Scott^41,83^, Thomas Seufferlein^138^, Jianxin Shi^166^, Albert Vernon Smith^167,168^, Joanna Smolonska^29,169^, Alice V. Stanton^170^, Valgerdur Steinthorsdottir^171^, Kathleen Stirrups^22,50^, Heather M. Stringham^1^, Johan Sundström^142^, Morris A. Swertz^29^, Amy J. Swift^99^, Ann-Christine Syvänen^16,172^, Sian-Tsung Tan^41,173^, Bamidele O. Tayo^174^, Barbara Thorand^120,175^, Gudmar Thorleifsson^171^, Jonathan P. Tyrer^176^, Hae-Won Uh^34,98^, Liesbeth Vandenput^177^, Frank C. Verhulst^178^, Sita H. Vermeulen^179,180^, Niek Verweij^57^, Judith M. Vonk^169^, Lindsay L. Waite^31^, Helen R. Warren^181^, Dawn Waterworth^182^, Michael N. Weedon^24^, Lynne R. Wilkens^54^, Christina Willenborg^183,184^, Tom Wilsgaard^185^, Mary K. Wojczynski^47^, Andrew Wong^186^, Alan F. Wright^126^, Qunyuan Zhang^47^, The LifeLines Cohort Study†, Eoin P. Brennan^187^, Murim Choi^188^, Zari Dastani^189^, Alexander W. Drong^18^, Per Eriksson^70^, Anders Franco-Cereceda^190^, Jesper R. Gådin^70^, Ali G. Gharavi^191^, Michael E. Goddard^192,193^, Robert E. Handsaker^6,7^, Jinyan Huang^194,195^, Fredrik Karpe^64,196^, Sekar Kathiresan^6,197^, Sarah Keildson^18^, Krzysztof Kiryluk^191^, Michiaki Kubo^198^, Jong-Young Lee^199^, Liming Liang^194,200^, Richard P. Lifton^201^, Baoshan Ma^194,202^, Steven A. McCarroll^6,7,162^, Amy J. McKnight^203^, Josine L. Min^146^, Miriam F. Moffatt^173^, Grant W. Montgomery^61^, Joanne M. Murabito^127,204^, George Nicholson^205,206^, Dale R. Nyholt^61,207^, Yukinori Okada^208,209^, John R. B. Perry^18,24,56^, Rajkumar Dorajoo^210^, Eva Reinmaa^14^, Rany M. Salem^5,6,7^, Niina Sandholm^211,212,213^, Robert A. Scott^9^, Lisette Stolk^34,60^, Atsushi Takahashi^208^, Toshihiro Tanaka^209,214,215^, Ferdinand M. van ’t Hooft^70^, Anna A. E. Vinkhuyzen^11^, Harm-Jan Westra^29^, Wei Zheng^216^, Krina T. Zondervan^18,217^, The ADIPOGen Consortium†, The AGEN-BMI Working Group†, The CARDIOGRAMplusC4D Consortium†, The CKDGen Consortium†, The GLGC†, The ICBP†, The MAGIC Investigators†, The MuTHER Consortium†, The MIGen Consortium†, The PAGE Consortium†, The ReproGen Consortium†, The GENIE Consortium†, The International Endogene Consortium†, Andrew C. Heath^218^, Dominique Arveiler^219^, Stephan J. L. Bakker^220^, John Beilby^87,221^, Richard N. Bergman^222^, John Blangero^92^, Pascal Bovet^223,224^, Harry Campbell^36^, Mark J. Caulfield^181^, Giancarlo Cesana^225^, Aravinda Chakravarti^44^, Daniel I. Chasman^163,226^, Peter S. Chines^99^, Francis S. Collins^99^, Dana C. Crawford^227,228^, L. Adrienne Cupples^127,229^, Daniele Cusi^230,231^, John Danesh^232^, Ulf de Faire^111^, Hester M. den Ruijter^76,233^, Anna F. Dominiczak^234^, Raimund Erbel^235^, Jeanette Erdmann^183,184^, Johan G. Eriksson^53,236,237^, Martin Farrall^18,48^, Stephan B. Felix^238,239^, Ele Ferrannini^240,241^, Jean Ferrières^242^, Ian Ford^243^, Nita G. Forouhi^9^, Terrence Forrester^244^, Oscar H. Franco^58,59^, Ron T. Gansevoort^220^, Pablo V. Gejman^245^, Christian Gieger^85^, Omri Gottesman^55^, Vilmundur Gudnason^167,168^, Ulf Gyllensten^135^, Alistair S. Hall^246^, Tamara B. Harris^109^, Andrew T. Hattersley^247^, Andrew A. Hicks^248^, Lucia A. Hindorff^249^, Aroon D. Hingorani^250^, Albert Hofman^58,59^, Georg Homuth^73^, G. Kees Hovingh^251^, Steve E. Humphries^252^, Steven C. Hunt^253^, Elina Hyppönen^254,255,256,257^, Thomas Illig^119,258^, Kevin B. Jacobs^3,79^, Marjo-Riitta Jarvelin^83,259,260,261,262,263^, Karl-Heinz Jöckel^63^, Berit Johansen^101^, Pekka Jousilahti^53^, J. Wouter Jukema^74,264,265^, Antti M. Jula^53^, Jaakko Kaprio^53,107,137^, John J. P. Kastelein^251^, Sirkka M. Keinanen-Kiukaanniemi^263,266^, Lambertus A. Kiemeney^179,267^, Paul Knekt^53^, Jaspal S. Kooner^41,173,268^, Charles Kooperberg^49^, Peter Kovacs^96,97^, Aldi T. Kraja^47^, Meena Kumari^269,270^, Johanna Kuusisto^271^, Timo A. Lakka^125,272,273^, Claudia Langenberg^9,269^, Loic Le Marchand^54^, Terho Lehtimäki^274^, Valeriya Lyssenko^275,276^, Satu Männistö^53^, André Marette^277,278^, Tara C. Matise^39^, Colin A. McKenzie^244^, Barbara McKnight^279^, Frans L. Moll^280^, Andrew D. Morris^106^, Andrew P. Morris^14,18,281^, Jeffrey C. Murray^282^, Mari Nelis^14^, Claes Ohlsson^177^, Albertine J. Oldehinkel^124^, Ken K. Ong^9,186^, Pamela A. F. Madden^218^, Gerard Pasterkamp^76^, John F. Peden^283^, Annette Peters^119,130,175^, Dirkje S. Postma^284^, Peter P. Pramstaller^248,285^, Jackie F. Price^36^, Lu Qi^13,25^, Olli T. Raitakari^286,287^, Tuomo Rankinen^288^, D. C. Rao^47,71,218^, Treva K. Rice^71,218^, Paul M. Ridker^163,226^, John D. Rioux^143,289^, Marylyn D. Ritchie^290^, Igor Rudan^36,291^, Veikko Salomaa^53^, Nilesh J. Samani^114,115^, Jouko Saramies^292^, Mark A. Sarzynski^288^, Heribert Schunkert^130,131^, Peter E. H. Schwarz^121,293^, Peter Sever^294^, Alan R. Shuldiner^295,296,297^, Juha Sinisalo^298^, Ronald P. Stolk^169^, Konstantin Strauch^85,153^, Anke Tönjes^96,97^, David-Alexandre Trégouët^299,300,301^, Angelo Tremblay^302^, Elena Tremoli^303^, Jarmo Virtamo^53^, Marie-Claude Vohl^278,304^, Uwe Völker^73,239^, Gérard Waeber^305^, Gonneke Willemsen^133^, Jacqueline C. Witteman^59^, M. Carola Zillikens^58,60^, Linda S. Adair^306^, Philippe Amouyel^307^, Folkert W. Asselbergs^250,264,308^, Themistocles L. Assimes^309^, Murielle Bochud^223,224^, Bernhard O. Boehm^310,311^, Eric Boerwinkle^312^, Stefan R. Bornstein^121^, Erwin P. Bottinger^55^, Claude Bouchard^288^, Stéphane Cauchi^80,81,82^, John C. Chambers^41,83,268^, Stephen J. Chanock^3^, Richard S. Cooper^174^, Paul I. W. de Bakker^77,313,314^, George Dedoussis^105^, Luigi Ferrucci^72^, Paul W. Franks^25,66,67^, Philippe Froguel^65,80,81,82^, Leif C. Groop^107,276^, Christopher A. Haiman^165^, Anders Hamsten^70^, Jennie Hui^87,221,315^, David J. Hunter^13,25,194^, Kristian Hveem^132^, Robert C. Kaplan^316^, Mika Kivimaki^269^, Diana Kuh^186^, Markku Laakso^271^, Yongmei Liu^317^, Nicholas G. Martin^61^, Winfried März^51,164,318^, Mads Melbye^309,319^, Andres Metspalu^14,147^, Susanne Moebus^63^, Patricia B. Munroe^181^, Inger Njølstad^185^, Ben A. Oostra^32,84,320^, Colin N. A. Palmer^106^, Nancy L. Pedersen^15^, Markus Perola^14,53,107^, Louis Pérusse^278,302^, Ulrike Peters^49^, Chris Power^257^, Thomas Quertermous^309^, Rainer Rauramaa^125,273^, Fernando Rivadeneira^58,59,60^, Timo E. Saaristo^321,322^, Danish Saleheen^232,323,324^, Naveed Sattar^325^, Eric E. Schadt^326^, David Schlessinger^155^, P. Eline Slagboom^34,35^, Harold Snieder^169^, Tim D. Spector^56^, Unnur Thorsteinsdottir^171,327^, Michael Stumvoll^96,97^, Jaakko Tuomilehto^53,328,329,330^, André G. Uitterlinden^58,59,60^, Matti Uusitupa^331,332^, Pim van der Harst^29,57,264^, Mark Walker^333^, Henri Wallaschofski^239,334^, Nicholas J. Wareham^9^, Hugh Watkins^18,48^, David R. Weir^26^, H-Erich Wichmann^335,336,337^, James F. Wilson^36^, Pieter Zanen^338^, Ingrid B. Borecki^47^, Panos Deloukas^22,50,339^, Caroline S. Fox^127^, Iris M. Heid^23,85^, Jeffrey R. O’Connell^295,296^, David P. Strachan^340^, Kari Stefansson^171,327^, Cornelia M. van Duijn^32,58,59,84^, Gonçalo R. Abecasis^1^, Lude Franke^29^, Timothy M. Frayling^24^, Mark I. McCarthy^18,64,341^, Peter M. Visscher^11,12^, André Scherag^63,342^, Cristen J. Willer^28,30,343^, Michael Boehnke^1^, Karen L. Mohlke^10^, Cecilia M. Lindgren^6,18^, Jacques S. Beckmann^20,21,344^, Inês Barroso^22,345,346^, Kari E. North^4,347^§, Erik Ingelsson^16,17,18^§, Joel N. Hirschhorn^5,6,7^§, Ruth J. F. Loos^9,55,147,348^§, & Elizabeth K. Speliotes^2^§

^1^Center for Statistical Genetics, Department of Biostatistics, University of Michigan, Ann Arbor, Michigan 48109, USA.

^2^Department of Internal Medicine, Division of Gastroenterology, and Department of Computational Medicine and Bioinformatics, University of Michigan, Ann Arbor, Michigan 48109, USA.

^3^Division of Cancer Epidemiology and Genetics, National Cancer Institute, National Institutes of Health, Bethesda, Maryland 20892, USA.

^4^Department of Epidemiology, University of North Carolina at Chapel Hill, Chapel Hill, North Carolina 27599, USA.

^5^Divisions of Endocrinology and Genetics and Center for Basic and Translational Obesity Research, Boston Children’s Hospital, Boston, Massachusetts 02115, USA.

^6^Broad Institute of the Massachusetts Institute of Technology and Harvard University, Cambridge, Massachusetts 02142, USA.

^7^Department of Genetics, Harvard Medical School, Boston, Massachusetts 02115, USA.

^8^Center for Biological Sequence Analysis, Department of Systems Biology, Technical University of Denmark, Lyngby 2800, Denmark.

^9^MRC Epidemiology Unit, University of Cambridge School of Clinical Medicine, Institute of Metabolic Science, Cambridge Biomedical Campus, Cambridge CB2 0QQ, UK.

^10^Department of Genetics, University of North Carolina, Chapel Hill, North Carolina 27599, USA.

^11^Queensland Brain Institute, The University of Queensland, Brisbane 4072, Australia.

^12^The University of Queensland Diamantina Institute, The Translation Research Institute, Brisbane 4012, Australia.

^13^Channing Division of Network Medicine, Department of Medicine, Brigham and Women's Hospital and Harvard Medical School, Boston, Massachusetts 02115, USA.

^14^Estonian Genome Center, University of Tartu, Tartu 51010, Estonia.

^15^Department of Medical Epidemiology and Biostatistics, Karolinska Institutet, Stockholm 17177, Sweden.

^16^Science for Life Laboratory, Uppsala University, Uppsala 75185, Sweden.

^17^Department of Medical Sciences, Molecular Epidemiology, Uppsala University, Uppsala 75185, Sweden.

^18^Wellcome Trust Centre for Human Genetics, University of Oxford, Oxford OX3 7BN, UK.

^19^Institute of Social and Preventive Medicine (IUMSP), Centre Hospitalier Universitaire Vaudois (CHUV), Lausanne 1010, Switzerland.

^20^Swiss Institute of Bioinformatics, Lausanne 1015, Switzerland.

^21^Department of Medical Genetics, University of Lausanne, Lausanne 1005, Switzerland.

^22^Wellcome Trust Sanger Institute, Hinxton, Cambridge CB10 1SA, UK.

^23^Department of Genetic Epidemiology, Institute of Epidemiology and Preventive Medicine, University of Regensburg, D-93053 Regensburg, Germany.

^24^Genetics of Complex Traits, University of Exeter Medical School, University of Exeter, Exeter EX1 2LU, UK.

^25^Department of Nutrition, Harvard School of Public Health, Boston, Massachusetts 02115, USA.

^26^Survey Research Center, Institute for Social Research, University of Michigan, Ann Arbor, Michigan 48104, USA.

^27^Department of Epidemiology, University of Michigan, Ann Arbor, Michigan 48109, USA.

^28^Department of Internal Medicine, Division of Cardiovascular Medicine, University of Michigan, Ann Arbor, Michigan 48109, USA.

^29^Department of Genetics, University Medical Center Groningen, University of Groningen, 9700 RB Groningen, The Netherlands.

^30^Department of Computational Medicine and Bioinformatics, University of Michigan, Ann Arbor, Michigan 48109, USA.

^31^HudsonAlpha Institute for Biotechnology, Huntsville, Alabama 35806, USA.

^32^Genetic Epidemiology Unit, Department of Epidemiology, Erasmus MC University Medical Center, 3015 GE Rotterdam, The Netherlands.

^33^Telethon Institute for Child Health Research, Centre for Child Health Research, The University of Western Australia, Perth, Western Australia 6008, Australia.

^34^Netherlands Consortium for Healthy Aging (NCHA), Leiden University Medical Center, Leiden 2300 RC, The Netherlands.

^35^Department of Molecular Epidemiology, Leiden University Medical Center, 2300 RC Leiden, The Netherlands.

^36^Centre for Population Health Sciences, University of Edinburgh, Teviot Place, Edinburgh EH8 9AG, UK.

^37^Kidney Epidemiology and Cost Center, University of Michigan, Ann Arbor, Michigan 48109, USA.

^38^Department of Statistics & Biostatistics, Rutgers University, Piscataway, New Jersey 08854, USA.

^39^Department of Genetics, Rutgers University, Piscataway, New Jersey 08854, USA.

^40^Department of Human Genetics, Leiden University Medical Center, 2333 ZC Leiden, The Netherlands.

^41^Ealing Hospital NHS Trust, Middlesex UB1 3HW, UK.

^42^Department of Gastroenterology and Hepatology, Imperial College London, London W2 1PG, UK.

^43^Institute of infectious Diseases, Southwest Hospital, Third Military Medical University, Chongqing, China.

^44^Center for Complex Disease Genomics, McKusick-Nathans Institute of Genetic Medicine, Johns Hopkins University School of Medicine, Baltimore, Maryland 21205, USA.

^45^Cardiology, Department of Specialties of Internal Medicine, Geneva University Hospital, Geneva 1211, Switzerland.

^46^Department of Epidemiology Research, Statens Serum Institut, Copenhagen DK-2300, Denmark.

^47^Department of Genetics, Washington University School of Medicine, St Louis, Missouri 63110, USA.

^48^Division of Cardiovacular Medicine, Radcliffe Department of Medicine, University of Oxford, Oxford OX3 9DU, UK.

^49^Division of Public Health Sciences, Fred Hutchinson Cancer Research Center, Seattle, Washington 98109, USA.

^50^William Harvey Research Institute, Barts and The London School of Medicine and Dentistry, Queen Mary University of London, London EC1M 6BQ, UK.

^51^Vth Department of Medicine (Nephrology, Hypertensiology, Endocrinology, Diabetology, Rheumatology), Medical Faculty of Mannheim, University of Heidelberg, D-68187 Mannheim, Germany.

^52^Department of Internal Medicine II, Ulm University Medical Centre, D-89081 Ulm, Germany.

^53^National Institute for Health and Welfare, FI-00271 Helsinki, Finland.

^54^Epidemiology Program, University of Hawaii Cancer Center, Honolulu, Hawaii 96813, USA.

^55^The Charles Bronfman Institute for Personalized Medicine, Icahn School of Medicine at Mount Sinai, New York, New York 10029, USA.

^56^Department of Twin Research and Genetic Epidemiology, King’s College London, London SE1 7EH, UK.

^57^Department of Cardiology, University Medical Center Groningen, University of Groningen, 9700RB Groningen, The Netherlands.

^58^Netherlands Consortium for Healthy Aging (NCHA), 3015GE Rotterdam, The Netherlands.

^59^Department of Epidemiology, Erasmus MC University Medical Center, 3015GE Rotterdam, The Netherlands.

^60^Department of Internal Medicine, Erasmus MC University Medical Center, 3015GE Rotterdam, The Netherlands.

^61^QIMR Berghofer Medical Research Institute, Brisbane, Queensland 4006, Australia.

^62^Laboratory of Neurogenetics, National Institute on Aging, National Institutes of Health, Bethesda, Maryland 20892, USA.

^63^Institute for Medical Informatics, Biometry and Epidemiology (IMIBE), University Hospital Essen, 45147 Essen, Germany.

^64^Oxford Centre for Diabetes, Endocrinology and Metabolism, University of Oxford, Oxford OX3 7LJ, UK.

^65^Department of Genomics of Common Disease, School of Public Health, Imperial College London, Hammersmith Hospital, London W12 0NN, UK.

^66^Department of Clinical Sciences, Genetic & Molecular Epidemiology Unit, Lund University Diabetes Center, Skåne University Hosptial, Malmö 205 02, Sweden.

^67^Department of Public Health and Clinical Medicine, Unit of Medicine, Umeå University, Umeå 901 87, Sweden.

^68^Department of Odontology, Umeå University, Umeå 901 85, Sweden.

^69^University of Eastern Finland, FI-70210 Kuopio, Finland.

^70^Atherosclerosis Research Unit, Center for Molecular Medicine, Department of Medicine, Karolinska Institutet, Stockholm 17176, Sweden.

^71^Division of Biostatistics, Washington University School of Medicine, St Louis, Missouri 63110, USA.

^72^Translational Gerontology Branch, National Institute on Aging, Baltimore, Maryland 21225, USA.

^73^Interfaculty Institute for Genetics and Functional Genomics, University Medicine Greifswald, D-17475 Greifswald, Germany.

^74^Department of Cardiology, Leiden University Medical Center, 2300 RC Leiden, The Netherlands.

^75^Department of Gerontology and Geriatrics, Leiden University Medical Center, 2300 RC Leiden, The Netherlands.

^76^Experimental Cardiology Laboratory, Division Heart and Lungs, University Medical Center Utrecht, 3584 CX Utrecht, The Netherlands.

^77^Department of Medical Genetics, University Medical Center Utrecht, 3584 CX Utrecht, The Netherlands.

^78^Department of Endocrinology, University of Groningen, University Medical Center Groningen, 9700 RB Groningen, The Netherlands.

^79^Core Genotyping Facility, SAIC-Frederick, Inc., NCI-Frederick, Frederick, Maryland 21702, USA.

^80^CNRS UMR 8199, F-59019 Lille, France.

^81^European Genomic Institute for Diabetes, F-59000 Lille, France.

^82^Université de Lille 2, F-59000 Lille, France.

^83^Department of Epidemiology and Biostatistics, Imperial College London, London W2 1PG, UK.

^84^Center for Medical Sytems Biology, 2300 RC Leiden, The Netherlands.

^85^Institute of Genetic Epidemiology, Helmholtz Zentrum München - German Research Center for Environmental Health, D-85764 Neuherberg, Germany.

^86^School of Health and Social Studies, Dalarna University, SE-791 88 Falun, Sweden.

^87^PathWest Laboratory Medicine of Western Australia, Nedlands, Western Australia 6009, Australia.

^88^Department of Haematology, University of Cambridge, Cambridge CB2 0PT, UK.

^89^NHS Blood and Transplant, Cambridge CB2 0PT, UK.

^90^Geriatric Unit, Azienda Sanitaria Firenze (ASF), 50125 Florence, Italy.

^91^USC-Office of Population Studies Foundation, Inc., University of San Carlos, Cebu City 6000, Philippines.

^92^Department of Genetics, Texas Biomedical Research Institute, San Antonio, Texas 78227, USA.

^93^Genomics Research Centre, Institute of Health and Biomedical Innovation, Queensland University of Technology, Brisbane, Queensland 4001, Australia.

^94^Department of Medical Sciences, Endocrinology, Diabetes and Metabolism, Uppsala University, Uppsala 75185, Sweden.

^95^Division of Endocrinology, Diabetes and Metabolism, Ulm University Medical Centre, D-89081 Ulm, Germany.

^96^Integrated Research and Treatment Center (IFB) Adiposity Diseases, University of Leipzig, D-04103 Leipzig, Germany.

^97^Department of Medicine, University of Leipzig, D-04103 Leipzig, Germany.

^98^Department of Medical Statistics and Bioinformatics, Leiden University Medical Center, 2300 RC Leiden, The Netherlands.

^99^Medical Genomics and Metabolic Genetics Branch, National Human Genome Research Institute, NIH, Bethesda, Maryland 20892, USA.

^100^LifeLines Cohort Study, University Medical Center Groningen, University of Groningen, 9700 RB Groningen, The Netherlands.

^101^Department of Biology, Norwegian University of Science and Technology, 7491 Trondheim, Norway.

^102^Department of Pediatrics, University of California Los Angeles, Torrance, California 90502, USA.

^103^Transgenomics Institute, Los Angeles Biomedical Research Institute, Torrance, California 90502, USA.

^104^Clinical Trial Service Unit and Epidemiological Studies Unit, Nuffield Department of Population Health, University of Oxford, Oxford OX3 7LF, UK.

^105^ Department of Dietetics-Nutrition, Harokopio University, 17671 Athens, Greece.

^106^Medical Research Institute, University of Dundee, Ninewells Hospital and Medical School, Dundee DD1 9SY, UK.

^107^Institute for Molecular Medicine, University of Helsinki, FI-00014 Helsinki, Finland.

^108^Analytic and Translational Genetics Unit, Massachusetts General Hospital and Harvard Medical School, Boston, Massachusetts 02114, USA.

^109^Laboratory of Epidemiology and Population Sciences, National Institute on Aging, NIH, Bethesda, Maryland 20892, USA.

^110^Department of Public Health and Caring Sciences, Geriatrics, Uppsala University, Uppsala 75185, Sweden.

^111^Division of Cardiovascular Epidemiology, Institute of Environmental Medicine, Karolinska Institutet, Stockholm, Sweden, Stockholm 17177, Sweden.

^112^Kaiser Permanente, Division of Research, Oakland, California 94612, USA.

^113^Service of Therapeutic Education for Diabetes, Obesity and Chronic Diseases, Geneva University Hospital, Geneva CH-1211, Switzerland.

^114^Department of Cardiovascular Sciences, University of Leicester, Glenfield Hospital, Leicester LE3 9QP, UK.

^115^National Institute for Health Research (NIHR) Leicester Cardiovascular Biomedical Research Unit, Glenfield Hospital, Leicester LE3 9QP, UK.

^116^Department of Nephrology, University Hospital Regensburg, D-93053 Regensburg, Germany.

^117^Department of Psychiatry and Psychotherapy, University Medicine Greifswald, HELIOS-Hospital Stralsund, D-17475 Greifswald, Germany.

^118^German Center for Neurodegenerative Diseases (DZNE), Rostock, Greifswald, D-17475 Greifswald, Germany.

^119^Research Unit of Molecular Epidemiology, Helmholtz Zentrum München - German Research Center for Environmental Health, D-85764 Neuherberg, Germany.

^120^German Center for Diabetes Research (DZD), 85764 Neuherberg, Germany.

^121^Department of Medicine III, University Hospital Carl Gustav Carus, Technische Universität Dresden, D-01307 Dresden, Germany.

^122^Institut inter Régional pour la Santé, Synergies, F-37520 La Riche, France.

^123^Department of Public Health and Clinical Medicine, Unit of Nutritional Research, Umeå University, Umeå 90187, Sweden.

^124^Department of Psychiatry, University of Groningen, University Medical Center Groningen, 9700RB Groningen, The Netherlands.

^125^Kuopio Research Institute of Exercise Medicine, FI-70100 Kuopio, Finland.

^126^MRC Human Genetics Unit, Institute of Genetics and Molecular Medicine, University of Edinburgh, Western General Hospital, Edinburgh EH4 2XU, UK.

^127^National Heart, Lung, and Blood Institute, the Framingham Heart Study, Framingham, Massachusetts 01702, USA.

^128^Department of Neurology, Boston University School of Medicine, Boston, Massachusetts 02118, USA.

^129^Faculty of Psychology and Education, VU University Amsterdam, 1081BT Amsterdam, The Netherlands.

^130^Deutsches Forschungszentrum für Herz-Kreislauferkrankungen (DZHK) (German Research Centre for Cardiovascular Research), Munich Heart Alliance, D-80636 Munich, Germany.

^131^Deutsches Herzzentrum München, Technische Universität München, D-80636 Munich, Germany.

^132^Department of Public Health and General Practice, Norwegian University of Science and Technology, Trondheim 7489, Norway.

^133^Biological Psychology, VU University Amsterdam, 1081BT Amsterdam, The Netherlands.

^134^Department of Pulmonary Physiology and Sleep Medicine, Nedlands, Western Australia 6009, Australia.

^135^School of Medicine and Pharmacology, University of Western Australia, Crawley 6009, Australia.

^136^Uppsala University, Department of Immunology, Genetics, Pathology, SciLifeLab, Rudbeck Laboratory, SE-751 85 Uppsala, Sweden.

^137^Hjelt Institute Department of Public Health, University of Helsinki, FI-00014 Helsinki, Finland.

^138^Department of Internal Medicine I, Ulm University Medical Centre, D-89081 Ulm, Germany.

^139^Finnish Institute of Occupational Health, FI-90100 Oulu, Finland.

^140^Division of Genetic Epidemiology, Department of Medical Genetics, Molecular and Clinical Pharmacology, Innsbruck Medical University, 6020 Innsbruck, Austria.

^141^Institute of Human Genetics, Helmholtz Zentrum München - German Research Center for Environmental Health, D-85764 Neuherberg, Germany.

^142^Department of Medical Sciences, Cardiovascular Epidemiology, Uppsala University, Uppsala 75185, Sweden.

^143^Montreal Heart Institute, Montreal, Quebec H1T 1C8, Canada.

^144^Institute for Community Medicine, University Medicine Greifswald, D-17475 Greifswald, Germany.

^145^The Genetics of Obesity and Related Metabolic Traits Program, The Icahn School of Medicine at Mount Sinai, New York, New York 10029, USA.

^146^School of Social and Community Medicine, University of Bristol, Bristol BS8 2BN, UK.

^147^Institute of Molecular and Cell Biology, University of Tartu, Tartu 51010, Estonia.

^148^Farr Institute of Health Informatics Research, University College London, London NW1 2DA, UK.

^149^The Center for Observational Research, Amgen, Inc., Thousand Oaks, California 91320, USA.

^150^Istituto di Ricerca Genetica e Biomedica (IRGB), Consiglio Nazionale delle Ricerche, Cagliari, Sardinia 09042, Italy.

^151^Center for Evidence-based Healthcare, University Hospital Carl Gustav Carus, Technische Universität Dresden, D-01307 Dresden, Germany.

^152^Department of Medicine I, University Hospital Grosshadern, Ludwig-Maximilians-Universität, D-81377 Munich, Germany.

^153^Institute of Medical Informatics, Biometry and Epidemiology, Chair of Genetic Epidemiology, Ludwig-Maximilians-Universität, D-81377 Munich, Germany.

^154^Department of Respiratory Medicine, Sir Charles Gairdner Hospital, Nedlands, Western Australia 6009, Australia.

^155^Laboratory of Genetics, National Institute on Aging, Baltimore, Maryland 21224, USA.

^156^Department of Genomics, Life & Brain Center, University of Bonn, 53127 Bonn, Germany.

^157^Institute of Human Genetics, University of Bonn, 53127 Bonn, Germany.

^158^Department of Epidemiology, University Medical Center Groningen, University of Groningen, 9700 RB Groningen, The Netherlands.

^159^Department of Epidemiology and Biostatistics, Institute for Research in Extramural Medicine, Institute for Health and Care Research, VU University Medical Center, 1081BT Amsterdam, The Netherlands.

^160^Department of Internal Medicine, Division of Endocrinology and Metabolism, Medical University of Graz, 8036 Graz, Austria.

^161^Institute of Physiology, University Medicine Greifswald, D-17495 Karlsburg, Germany.

^162^Stanley Center for Psychiatric Research, Broad Institute of MIT and Harvard, Cambridge, Massachusetts 02142, USA.

^163^Division of Preventive Medicine, Brigham and Women's Hospital, Boston, Massachusetts 02215, USA.

^164^Clinical Institute of Medical and Chemical Laboratory Diagnostics, Medical University of Graz, Graz 8036, Austria.

^165^Department of Preventive Medicine, Keck School of Medicine, University of Southern California, Los Angeles, California 90089, USA.

^166^National Cancer Institute, Bethesda, Maryland 20892, USA.

^167^Icelandic Heart Association, Kopavogur 201, Iceland.

^168^University of Iceland, Reykjavik 101, Iceland.

^169^Department of Epidemiology, University Medical Center Groningen, University of Groningen, 9700 RB Groningen, The Netherlands.

^170^Molecular & Cellular Therapeutics, Royal College of Surgeons in Ireland, 123 St. Stephen’s Green, Dublin 2, Ireland.

^171^deCODE Genetics, Amgen Inc., Reykjavik 101, Iceland.

^172^Department of Medical Sciences, Molecular Medicine, Uppsala University, Uppsala 75144, Sweden.

^173^National Heart and Lung Institute, Imperial College London, London SW3 6LY, UK.

^174^Department of Public Health Sciences, Stritch School of Medicine, Loyola University of Chicago, Maywood, Illinois 61053, USA.

^175^Institute of Epidemiology II, Helmholtz Zentrum München - German Research Center for Environmental Health, Neuherberg, Germany, D-85764 Neuherberg, Germany.

^176^Department of Oncology, University of Cambridge, Cambridge CB2 0QQ, UK.

^177^Centre for Bone and Arthritis Research, Department of Internal Medicine and Clinical Nutrition, Institute of Medicine, Sahlgrenska Academy, University of Gothenburg, Gothenburg 413 45, Sweden.

^178^Department of Child and Adolescent Psychiatry/Psychology, Erasmus MC University Medical Centre, 3000 CB Rotterdam, The Netherlands.

^179^Department for Health Evidence, Radboud University Medical Centre, 6500 HB Nijmegen, The Netherlands.

^180^Department of Genetics, Radboud University Medical Centre, 6500 HB Nijmegen, The Netherlands.

^181^Department of Clinical Pharmacology, William Harvey Research Institute, Barts and The London School of Medicine and Dentistry, Queen Mary University of London, London EC1M 6BQ, UK.

^182^Genetics, GlaxoSmithKline, King of Prussia, Pennsylvania 19406, USA.

^183^German Center for Cardiovascular Research, partner site Hamburg/Lubeck/Kiel, 23562 Lubeck, Germany.

^184^Institut für Integrative und Experimentelle Genomik, Universität zu Lübeck, D-23562 Lübeck, Germany.

^185^Department of Community Medicine, Faculty of Health Sciences, UiT The Arctic University of Norway, 9037 Tromsø, Norway.

^186^MRC Unit for Lifelong Health and Ageing at University College London, London WC1B 5JU, UK.

^187^Diabetes Complications Research Centre, Conway Institute, School of Medicine and Medical Sciences, University College Dublin, Dublin 4, Ireland.

^188^Department of Biomedical Sciences, Seoul National University College of Medicine, Seoul, Korea.

^189^Lady Davis Institute, Departments of Human Genetics, Epidemiology and Biostatistics, McGill University, Montréal, Québec H3T1E2, Canada.

^190^Cardiothoracic Surgery Unit, Department of Molecular Medicine and Surgery, Karolinska Institutet, Stockholm 17176, Sweden.

^191^Department of Medicine, Columbia University College of Physicians and Surgeons, New York 10032, USA.

^192^Biosciences Research Division, Department of Primary Industries, Victoria 3083, Australia.

^193^Department of Food and Agricultural Systems, University of Melbourne, Victoria 3010, Australia.

^194^Department of Epidemiology, Harvard School of Public Health, Boston, Massachusetts 02115, USA.

^195^State Key Laboratory of Medical Genomics, Shanghai Institute of Hematology, Rui Jin Hospital Affiliated with Shanghai Jiao Tong University School of Medicine, Shanghai, China.

^196^NIHR Oxford Biomedical Research Centre, OUH Trust, Oxford OX3 7LE, UK.

^197^Cardiovascular Research Center, Massachusetts General Hospital, Harvard Medical School, Boston, Massachusetts, USA.

^198^Laboratory for Genotyping Development, RIKEN Center for Integrative Medical Sciences, Yokohama 230-0045, Japan.

^199^Center for Genome Science, National Institute of Health, Chungcheongbuk-do, Chungbuk 363–951, Republic of Korea.

^200^Harvard School of Public Health, Department of Biostatistics, Harvard University, Boston, Massachusetts 2115, USA.

^201^Department of Genetics, Howard Hughes Medical Institute, Yale University School of Medicine, New Haven, New Haven, Connecticut 06520, USA.

^202^College of Information Science and Technology, Dalian Maritime University, Dalian, Liaoning 116026, China.

^203^Nephrology Research, Centre for Public Health, Queen’s University of Belfast, Belfast, County Down BT9 7AB, UK.

^204^Section of General Internal Medicine, Boston University School of Medicine, Boston, Massachusetts 02118, USA.

^205^Department of Statistics, University of Oxford, 1 South Parks Road, Oxford OX1 3TG, UK.

^206^MRC Harwell, Harwell Science and Innovation Campus, Harwell OX11 0QG, UK.

^207^Institute of Health and Biomedical Innovation, Queensland University of Technology, Brisbane, Queensland 4059, Australia.

^208^Laboratory for Statistical Analysis, RIKEN Center for Integrative Medical Sciences, Yokohama 230-0045, Japan.

^209^Department of Human Genetics and Disease Diversity, Graduate School of Medical and Dental Sciences, Tokyo Medical and Dental University, 113-8510 Tokyo, Japan.

^210^Genome Institute of Singapore, Agency for Science, Technology and Research, 138672 Singapore.

^211^Department of Biomedical Engineering and Computational Science, Aalto University School of Science, Helsinki FI-00076, Finland.

^212^Department of Medicine, Division of Nephrology, Helsinki University Central Hospital, FI-00290 Helsinki, Finland.

^213^Folkhälsan Institute of Genetics, Folkhälsan Research Center, FI-00290 Helsinki, Finland.

^214^Laboratory for Cardiovascular Diseases, RIKEN Center for Integrative Medical Sciences, Yokohama 230-0045, Japan.

^215^Division of Disease Diversity, Bioresource Research Center, Tokyo Medical and Dental University, 113-8510 Tokyo, Japan.

^216^Division of Epidemiology, Department of Medicine; Vanderbilt Epidemiology Center; and Vanderbilt-Ingram Cancer Center, Vanderbilt University Medical Center, Nashville, Tennessee 37075, USA.

^217^Nuffield Department of Obstetrics & Gynaecology, University of Oxford, Oxford OX3 7BN, UK.

^218^Department of Psychiatry, Washington University School of Medicine, St Louis, Missouri 63110, USA.

^219^Department of Epidemiology and Public Health, EA3430, University of Strasbourg, Faculty of Medicine, Strasbourg, France.

^220^Department of Internal Medicine, University Medical Center Groningen, University of Groningen, 9700RB Groningen, The Netherlands.

^221^Pathology and Laboratory Medicine, The University of Western Australia, Perth, Western Australia 6009, Australia.

^222^Cedars-Sinai Diabetes and Obesity Research Institute, Los Angeles, California 90048, USA.

^223^Institute of Social and Preventive Medicine (IUMSP), Centre Hospitalier Universitaire Vaudois and University of Lausanne, 1010 Lausanne, Switzerland.

^224^Ministry of Health, Victoria, Republic of Seychelles.

^225^University of Milano, Bicocca, 20126, Italy.

^226^Harvard Medical School, Boston, Massachusetts 02115, USA.

^227^Center for Human Genetics Research, Vanderbilt University Medical Center, Nashville, Tennessee 37203, USA.

^228^Department of Molecular Physiology and Biophysics, Vanderbilt University, Nashville, Tennessee 37232, USA.

^229^Department of Biostatistics, Boston University School of Public Health, Boston, Massachusetts 02118, USA.

^230^Department of Health Sciences, University of Milano, I 20142, Italy.

^231^Fondazione Filarete, Milano I 20139, Italy.

^232^Department of Public Health and Primary Care, University of Cambridge, Cambridge CB1 8RN, UK.

^233^Julius Center for Health Sciences and Primary Care, University Medical Center Utrecht, 3584 CX Utrecht, The Netherlands.

^234^Institute of Cardiovascular and Medical Sciences, College of Medical, Veterinary and Life Sciences, University of Glasgow, Glasgow G12 8TA, UK.

^235^Clinic of Cardiology, West-German Heart Centre, University Hospital Essen, 45147 Essen, Germany.

^236^Department of General Practice and Primary Health Care, University of Helsinki, FI-00290 Helsinki, Finland.

^237^Unit of General Practice, Helsinki University Central Hospital, Helsinki 00290, Finland.

^238^Department of Internal Medicine B, University Medicine Greifswald, D-17475 Greifswald, Germany.

^239^DZHK (Deutsches Zentrum für Herz-Kreislaufforschung – German Centre for Cardiovascular Research), partner site Greifswald, D-17475 Greifswald, Germany.

^240^Department of Internal Medicine, University of Pisa, 56100 Pisa, Italy.

^241^National Research Council Institute of Clinical Physiology, University of Pisa, 56124 Pisa, Italy.

^242^Department of Cardiology, Toulouse University School of Medicine, Rangueil Hospital, 31400 Toulouse, France.

^243^Robertson Center for Biostatistics, University of Glasgow, Glasgow G12 8QQ, UK.

^244^UWI Solutions for Developing Countries, The University of the West Indies, Mona, Kingston 7, Jamaica.

^245^NorthShore University HealthSystem, Evanston, IL 60201, University of Chicago, Chicago, Illinois, USA.

^246^Leeds MRC Medical Bioinformatics Centre, University of Leeds, Leeds LS2 9LU, UK.

^247^Institute of Biomedical & Clinical Science, University of Exeter, Barrack Road, Exeter EX2 5DW, UK.

^248^Center for Biomedicine, European Academy Bozen, Bolzano (EURAC), Bolzano 39100, Italy (affiliated institute of the University of Lübeck, D-23562 Lübeck, Germany).

^249^Division of Genomic Medicine, National Human Genome Research Institute, National Institutes of Health, Bethesda, Maryland 20892, USA.

^250^Institute of Cardiovascular Science, University College London, London WC1E 6BT, UK.

^251^Department of Vascular Medicine, Academic Medical Center, 1105 AZ Amsterdam, The Netherlands. ^252^Centre for Cardiovascular Genetics, Institute Cardiovascular Sciences, University College London, London WC1E 6JJ, UK.

^253^Cardiovascular Genetics Division, Department of Internal Medicine, University of Utah, Salt Lake City, Utah 84108, USA.

^254^Sansom Institute for Health Research, University of South Australia, Adelaide 5000, South Australia, Australia.

^255^School of Population Health, University of South Australia, Adelaide 5000, South Australia, Australia.

^256^South Australian Health and Medical Research Institute, Adelaide, South Australia 5000, Australia.

^257^Population, Policy, and Practice, University College London Institute of Child Health, London WC1N 1EH, UK.

^258^Hannover Unified Biobank, Hannover Medical School, Hannover, D-30625 Hannover, Germany.

^259^National Institute for Health and Welfare, FI-90101 Oulu, Finland.

^260^MRC Health Protection Agency (HPA) Centre for Environment and Health, School of Public Health, Imperial College London, London W2 1PG, UK.

^261^Unit of Primary Care, Oulu University Hospital, FI-90220 Oulu, Finland.

^262^Biocenter Oulu, University of Oulu, FI-90014 Oulu, Finland.

^263^Institute of Health Sciences, University of Oulu, FI-90014 Oulu, Finland.

^264^Durrer Center for Cardiogenetic Research, Interuniversity Cardiology Institute Netherlands (ICIN), 3501 DG Utrecht, The Netherlands.

^265^Interuniversity Cardiology Institute of the Netherlands (ICIN), 3501 DG Utrecht, The Netherlands.

^266^Unit of Primary Health Care/General Practice, Oulu University Hospital, FI-90220 Oulu, Finland.

^267^Department of Urology, Radboud University Medical Centre, 6500 HB Nijmegen, The Netherlands.

^268^Imperial College Healthcare NHS Trust, London W12 0HS, UK.

^269^Department of Epidemiology and Public Health, University College London, London WC1E 6BT, UK.

^270^Department of Biological and Social Epidemiology, University of Essex, Wivenhoe Park, Colchester, Essex CO4 3SQ, UK.

^271^Department of Medicine, Kuopio University Hospital and University of Eastern Finland, FI-70210 Kuopio, Finland.

^272^Department of Physiology, Institute of Biomedicine, University of Eastern Finland, Kuopio Campus, FI-70211 Kuopio, Finland.

^273^Department of Clinical Physiology and Nuclear Medicine, Kuopio University Hospital and University of Eastern Finland, FI-70210 Kuopio, Finland.

^274^Department of Clinical Chemistry, Fimlab Laboratories and School of Medicine University of Tampere, FI-33520 Tampere, Finland.

^275^Steno Diabetes Center A/S, Gentofte DK-2820, Denmark.

^276^Lund University Diabetes Centre and Department of Clinical Science, Diabetes & Endocrinology Unit, Lund University, Malmö 221 00, Sweden.

^277^Institut Universitaire de Cardiologie et de Pneumologie de Québec, Faculty of Medicine, Laval University, Quebec, QC G1V 0A6, Canada.

^278^Institute of Nutrition and Functional Foods, Laval University, Quebec, QC G1V 0A6, Canada.

^279^Department of Biostatistics, University of Washington, Seattle, Washington 98195, USA.

^280^Department of Surgery, University Medical Center Utrecht, 3584 CX Utrecht, The Netherlands.

^281^Department of Biostatistics, University of Liverpool, Liverpool L69 3GA, UK.

^282^Department of Pediatrics, University of Iowa, Iowa City, Iowa 52242, USA.

^283^Illumina, Inc, Little Chesterford, Cambridge CB10 1XL, UK.

^284^University of Groningen, University Medical Center Groningen, Department of Pulmonary Medicine and Tuberculosis, Groningen, The Netherlands.

^285^Department of Neurology, General Central Hospital, Bolzano 39100, Italy.

^286^Department of Clinical Physiology and Nuclear Medicine, Turku University Hospital, FI-20521 Turku, Finland.

^287^Research Centre of Applied and Preventive Cardiovascular Medicine, University of Turku, FI-20521 Turku, Finland.

^288^Human Genomics Laboratory, Pennington Biomedical Research Center, Baton Rouge, Louisiana 70808, USA.

^289^Université de Montréal, Montreal, Quebec H1T 1C8, Canada.

^290^Center for Systems Genomics, The Pennsylvania State University, University Park, Pennsylvania 16802, USA.

^291^Croatian Centre for Global Health, Faculty of Medicine, University of Split, 21000 Split, Croatia.

^292^South Carelia Central Hospital, 53130 Lappeenranta, Finland.

^293^Paul Langerhans Institute Dresden, German Center for Diabetes Research (DZD), 01307 Dresden, Germany.

^294^International Centre for Circulatory Health, Imperial College London, London W2 1PG, UK.

^295^Division of Endocrinology, Diabetes and Nutrition, University of Maryland School of Medicine, Baltimore, Maryland 21201, USA.

^296^Program for Personalized and Genomic Medicine, University of Maryland School of Medicine, Baltimore, Maryland 21201, USA.

^297^Geriatric Research and Education Clinical Center, Vetrans Administration Medical Center, Baltimore, Maryland 21201, USA.

^298^Helsinki University Central Hospital Heart and Lung Center, Department of Medicine, Helsinki University Central Hospital, FI-00290 Helsinki, Finland.

^299^Sorbonne Universités, UPMC Univ Paris 06, UMR S 1166, F-75013 Paris, France.

^300^INSERM, UMR S 1166, Team Genomics and Physiopathology of Cardiovascular Diseases, F-75013 Paris, France.

^301^Institute for Cardiometabolism And Nutrition (ICAN), F-75013 Paris, France.

^302^Department of Kinesiology, Laval University, Quebec QC G1V 0A6, Canada.

^303^Dipartimento di Scienze Farmacologiche e Biomolecolari, Università di Milano & Centro Cardiologico Monzino, Instituto di Ricovero e Cura a Carattere Scientifico, Milan 20133, Italy.

^304^Department of Food Science and Nutrition, Laval University, Quebec QC G1V 0A6, Canada.

^305^Department of Internal Medicine, University Hospital (CHUV) and University of Lausanne, Lausanne 1011, Switzerland.

^306^Department of Nutrition, University of North Carolina, Chapel Hill, North Carolina 27599, USA.

^307^Institut Pasteur de Lille; INSERM, U744; Université de Lille 2; F-59000 Lille, France.

^308^Department of Cardiology, Division Heart and Lungs, University Medical Center Utrecht, 3584 CX Utrecht, The Netherlands.

^309^Department of Medicine, Stanford University School of Medicine, Palo Alto, California 94304, USA.

^310^Lee Kong Chian School of Medicine, Imperial College London and Nanyang Technological University, Singapore, 637553 Singapore, Singapore.

^311^Department of Internal Medicine I, Ulm University Medical Centre, D-89081 Ulm, Germany.

^312^Health Science Center at Houston, University of Texas, Houston, Texas 77030, USA.

^313^Department of Medicine, Division of Genetics, Brigham and Women’s Hospital, Harvard Medical School, Boston, Massachusetts 02115, USA.

^314^Department of Epidemiology, University Medical Center Utrecht, 3584 CX Utrecht, The Netherlands.

^315^School of Population Health, The University of Western Australia, Nedlands, Western Australia 6009, Australia.

^316^Albert Einstein College of Medicine. Department of Epidemiology and Population Health, Belfer 1306, New York 10461, USA.

^317^Center for Human Genetics, Division of Public Health Sciences, Wake Forest School of Medicine, Winston-Salem, North Carolina 27157, USA.

^318^Synlab Academy, Synlab Services GmbH, 68163 Mannheim, Germany.

^319^Department of Clinical Medicine, Copenhagen University, 2200 Copenhagen, Denmark.

^320^Department of Clinical Genetics, Erasmus MC University Medical Center, 3000 CA Rotterdam, The Netherlands.

^321^Finnish Diabetes Association, Kirjoniementie 15, FI-33680 Tampere, Finland.

^322^Pirkanmaa Hospital District, FI-33521 Tampere, Finland.

^323^Center for Non-Communicable Diseases, Karatchi, Pakistan.

^324^Department of Medicine, University of Pennsylvania, Philadelphia, Pennsylvania 19104, USA.

^325^BHF Glasgow Cardiovascular Research Centre, Division of Cardiovascular and Medical Sciences, University of Glasgow, Glasgow G12 8TA, UK.

^326^Icahn Institute for Genomics and Multiscale Biology, Icahn School of Medicine at Mount Sinai, New York, New York 10580, USA.

^327^Faculty of Medicine, University of Iceland, Reykjavik 101, Iceland.

^328^Institute for Health Research, University Hospital of La Paz (IdiPaz), Madrid 28046, Spain.

^329^Diabetes Research Group, King Abdulaziz University, 21589 Jeddah, Saudi Arabia.

^330^Centre for Vascular Prevention, Danube-University Krems, 3500 Krems, Austria.

^331^Department of Public Health and Clinical Nutrition, University of Eastern Finland, FI-70210 Kuopio, Finland.

^332^Research Unit, Kuopio University Hospital, FI-70210 Kuopio, Finland.

^333^Institute of Cellular Medicine, Newcastle University, Newcastle NE1 7RU, UK.

^334^Institute of Clinical Chemistry and Laboratory Medicine, University Medicine Greifswald, D-17475 Greifswald, Germany.

^335^Institute of Medical Informatics, Biometry and Epidemiology, Chair of Epidemiology, Ludwig-Maximilians-Universität, D-85764 Munich, Germany.

^336^Klinikum Grosshadern, D-81377 Munich, Germany.

^337^Institute of Epidemiology I, Helmholtz Zentrum München - German Research Center for Environmental Health, Neuherberg, Germany, D-85764 Neuherberg, Germany.

^338^Department of Pulmonology, University Medical Center Utrecht, 3584 CX Utrecht, The Netherlands.

^339^Princess Al-Jawhara Al-Brahim Centre of Excellence in Research of Hereditary Disorders (PACER-HD), King Abdulaziz University, 21589 Jeddah, Saudi Arabia.

^340^Division of Population Health Sciences & Education, St George’s, University of London, London SW17 0RE, UK.

^341^Oxford NIHR Biomedical Research Centre, Oxford University Hospitals NHS Trust, Oxford OX3 7LJ, UK.

^342^Clinical Epidemiology, Integrated Research and Treatment Center, Center for Sepsis Control and Care (CSCC), Jena University Hospital, 07743 Jena, Germany.

^343^Department of Human Genetics, University of Michigan, Ann Arbor, Michigan 48109, USA.

^344^Service of Medical Genetics, CHUV University Hospital, 1011 Lausanne, Switzerland

^345^University of Cambridge Metabolic Research Laboratories, Institute of Metabolic Science, Addenbrooke’s Hospital, Cambridge CB2 OQQ, UK.

^346^NIHR Cambridge Biomedical Research Centre, Institute of Metabolic Science, Addenbrooke’s Hospital, Cambridge CB2 OQQ, UK.

^347^Carolina Center for Genome Sciences, University of North Carolina at Chapel Hill, Chapel Hill, North Carolina 27599, USA.

^348^The Mindich Child Health and Development Institute, Icahn School of Medicine at Mount Sinai, New York, New York 10029, USA.
